# *Pseudomonas aeruginosa* Lifestyle: A Paradigm for Adaptation, Survival, and Persistence

**DOI:** 10.3389/fcimb.2017.00039

**Published:** 2017-02-15

**Authors:** M. Fata Moradali, Shirin Ghods, Bernd H. A. Rehm

**Affiliations:** Institute of Fundamental Sciences, Massey UniversityPalmerston North, New Zealand

**Keywords:** *Pseudomonas aeruginosa*, virulence, biofilm, antibiotic resistance, persistence

## Abstract

*Pseudomonas aeruginosa* is an opportunistic pathogen affecting immunocompromised patients. It is known as the leading cause of morbidity and mortality in cystic fibrosis (CF) patients and as one of the leading causes of nosocomial infections. Due to a range of mechanisms for adaptation, survival and resistance to multiple classes of antibiotics, infections by *P. aeruginosa* strains can be life-threatening and it is emerging worldwide as public health threat. This review highlights the diversity of mechanisms by which *P. aeruginosa* promotes its survival and persistence in various environments and particularly at different stages of pathogenesis. We will review the importance and complexity of regulatory networks and genotypic-phenotypic variations known as adaptive radiation by which *P. aeruginosa* adjusts physiological processes for adaptation and survival in response to environmental cues and stresses. Accordingly, we will review the central regulatory role of quorum sensing and signaling systems by nucleotide-based second messengers resulting in different lifestyles of *P. aeruginosa*. Furthermore, various regulatory proteins will be discussed which form a plethora of controlling systems acting at transcriptional level for timely expression of genes enabling rapid responses to external stimuli and unfavorable conditions. Antibiotic resistance is a natural trait for *P. aeruginosa* and multiple mechanisms underlying different forms of antibiotic resistance will be discussed here. The importance of each mechanism in conferring resistance to various antipseudomonal antibiotics and their prevalence in clinical strains will be described. The underlying principles for acquiring resistance leading pan-drug resistant strains will be summarized. A future outlook emphasizes the need for collaborative international multidisciplinary efforts to translate current knowledge into strategies to prevent and treat *P. aeruginosa* infections while reducing the rate of antibiotic resistance and avoiding the spreading of resistant strains.

## Introduction

*Pseudomonas aeruginosa* is a Gram-negative and ubiquitous environmental bacterium. It is an opportunistic human pathogen capable of causing a wide array of life-threatening acute and chronic infections, particularly in patients with compromised immune defense. It has been of particular importance since it is the main cause of morbidity and mortality in cystic fibrosis (CF) patients and one of the leading nosocomial pathogens affecting hospitalized patients while being intrinsically resistant to a wide range of antibiotics.

*P. aeruginosa* strains possess large genomes (~5–7 Mbp). Their metabolic capacity is extensive as exemplified by their ability to produce multiple secondary metabolites and polymers as well as their ability to use various carbon sources and electron acceptors. The repertoire of *P. aeruginosa* genes which are substantially conserved suggest the highest proportion of regulatory genes and networks observed in known bacterial genomes and is foundational to respond and adapt to diverse environments (Stover et al., 2000; Mathee et al., 2008; Frimmersdorf et al., 2010). The ubiquitous presence of *P. aeruginosa* as well as its prevalence and persistence in clinical settings including intrinsic resistance to therapeutics are attributed to its extraordinary capability of survival by recruiting an arsenal of responsive mechanisms.

In the present review, we have attempted to summarize the diversity of these mechanisms causing the versatility of *P. aeruginosa* to adapt and thrive in unfavorable conditions particularly during pathogenesis. To this end, we will describe the clinical importance of *P. aeruginosa* followed by the well-characterized and most recent findings about key strategic adaptation mechanisms including quorum sensing (QS), motility-sessility switch, biofilm formation, antibiotic resistance mechanisms, adaptive radiation for persistence, stringent response and persisters, and the CRISPR-Cas systems. Recent findings on adaptive mechanisms will be set into context of the overall physiology of *P. aeruginosa* by also depicting on future research needs.

## Clinical importance

The CF patients suffer from a multisystem disease due to inheritable genetic defects in the CF transmembrane conductance regulator (*CFTR*) gene. However, the recurrence of bacterial infections in the abnormal mucus layers is the main cause of morbidity and mortality of CF patients (Khan et al., 1995; Rosenfeld et al., 2001). The CFTR regulator is responsible for regulating the transport of electrolytes and chloride across epithelial cell membranes to maintain normal mucus properties and homeostasis. Therefore, the loss of function of the CFTR protein results in abnormally thick, dehydrated and sticky mucus layers in the lung (Flume and Van Devanter, 2012). Hence, the CF patients are largely susceptible to respiratory infections by *P. aeruginosa* from infancy. When they are under a year old, almost 30% of CF infants can acquire initial *P. aeruginosa* strains from the environment leading to acute infections. This rate increases to about 50% by the age of 3 years while mucoid phenotypes causing chronic infections have been reported emerging at the age of 3 to16 years (median of 13 years) (Rehm et al., 1994; da Silva et al., 2013; Jones et al., 2016). *P. aeruginosa* will adapt to CF airways and persist as overwhelming, predominant and ineradicable infections to the end of patients' life in almost 70% of adults (Döring et al., 2000).

Furthermore, *P. aeruginosa* is also largely associated with hospital acquired infections including ventilator-associated pneumonia, central line-associated bloodstream infection, urinary catheter-related infection, and surgical/transplantation infections (Cardo et al., 2004; Nathwani et al., 2014; Trubiano and Padiglione, 2015). The International Nosocomial Infection Control Consortium reported that *P. aeruginosa* nosocomial infections have become a worldwide healthcare issue (Rosenthal et al., 2016). A cohort study reported that *P. aeruginosa* had the highest burden of healthcare-acquired infections in European intensive care units (Lambert et al., 2011). In the United States healthcare-associated *P. aeruginosa* infections were estimated to contribute to 51,000 cases each year (Eurosurveillance Editorial Team, 2013). *P. aeruginosa* is prevalent in healthcare settings because it is a common companion of patients under medical care and also it can survive on abiotic and biotic surfaces such as medical equipment resisting disinfection methods while being transmissible from patient-to-patient (Russotto et al., 2015).

*P. aeruginosa* infections are becoming more difficult to treat because this bacterium is naturally resistant to many antibiotics and the number of multidrug- and pan-drug-resistant strains is increasing worldwide. Strains have been reported which are resistant to almost all class of commonly used antibiotics including aminoglycosides, cephalosporins, fluoroquinolones, and carbapenems (Hancock and Speert, 2000; Poole, 2011; Eurosurveillance Editorial Team, 2013). In the United States about 13% of *P. aeruginosa* infections are caused by multidrug resistant strains (Eurosurveillance Editorial Team, 2013).

*P. aeruginosa* utilizes sophisticated genotypic events to support various phenotypes and molecular mechanisms required for survival during pathogenesis and antibiotic treatment.

Therefore, at initial stages of CF lung colonization, a large number of virulence and intrinsic antibiotic resistance mechanisms mediate survival. After infection, bacteria are exposed to inflammatory responses including oxidative stress followed by treatment with antibiotics (Furukawa et al., 2006; Turner et al., 2014). These environmental stress factors induce the expression of different sets of genes enabling *P. aeruginosa* to adapt and switch to persisting and resistant phenotypes, while becoming less virulent, such as upon formation of mucoid biofilms (MacDougall et al., 2005; Poole, 2012; Gellatly and Hancock, 2013). Due to the existence of an arsenal of molecular mechanisms conferring resistance to multiple classes of antibiotics, therapeutic options are increasingly limited for treatment of infections, while the number of infection incidences and multi-drug resistance strains are increasing.

## Central regulatory role of quorum sensing (QS) for virulence and adaptation

Communication between individual cells using specific chemical signals is a well-known capability of bacteria and is called quorum sensing. Indeed, QS controls social behavior of bacteria by multiple interconnected signaling pathways (LaSarre and Federle, 2013). It allows bacterial communities to regulate a variety of biological processes important for bacterial adaptation and survival. Basically, this phenomenon relies on regulating the expression of specific sets of genes in response to a critical threshold of signaling molecules known as autoinducers (AIs). QS will mediate population density dependent collective responses and is therefore beneficial for community survival. A study showed that cells' responses to QS signals and the corresponding gene expression profile is heterogeneous within a given community leading to increasing fitness and chance of survival (Grote et al., 2015).

During pathogenesis *P. aeruginosa* QS plays a critical role for survival and colonization by coordinating phenotypic alterations at early stages of infection i.e., after attachment (González and Keshavan, 2006). The progress of acute to chronic infection is critically influenced by QS-dependent gene expression. More than 10% of *P. aeruginosa* genes are regulated by QS. These genes are mainly involved in virulence factor production, motility, motility-sessility switch and biofilm development, antibiotic resistance mechanisms and the adjustment of metabolic pathways for stress responses (Venturi, 2006; Williams and Camara, 2009; Barr et al., 2015). The role of QS in each physiological adaptation will be discussed below.

### Molecular mechanisms underlying QS

**Figure 1:**
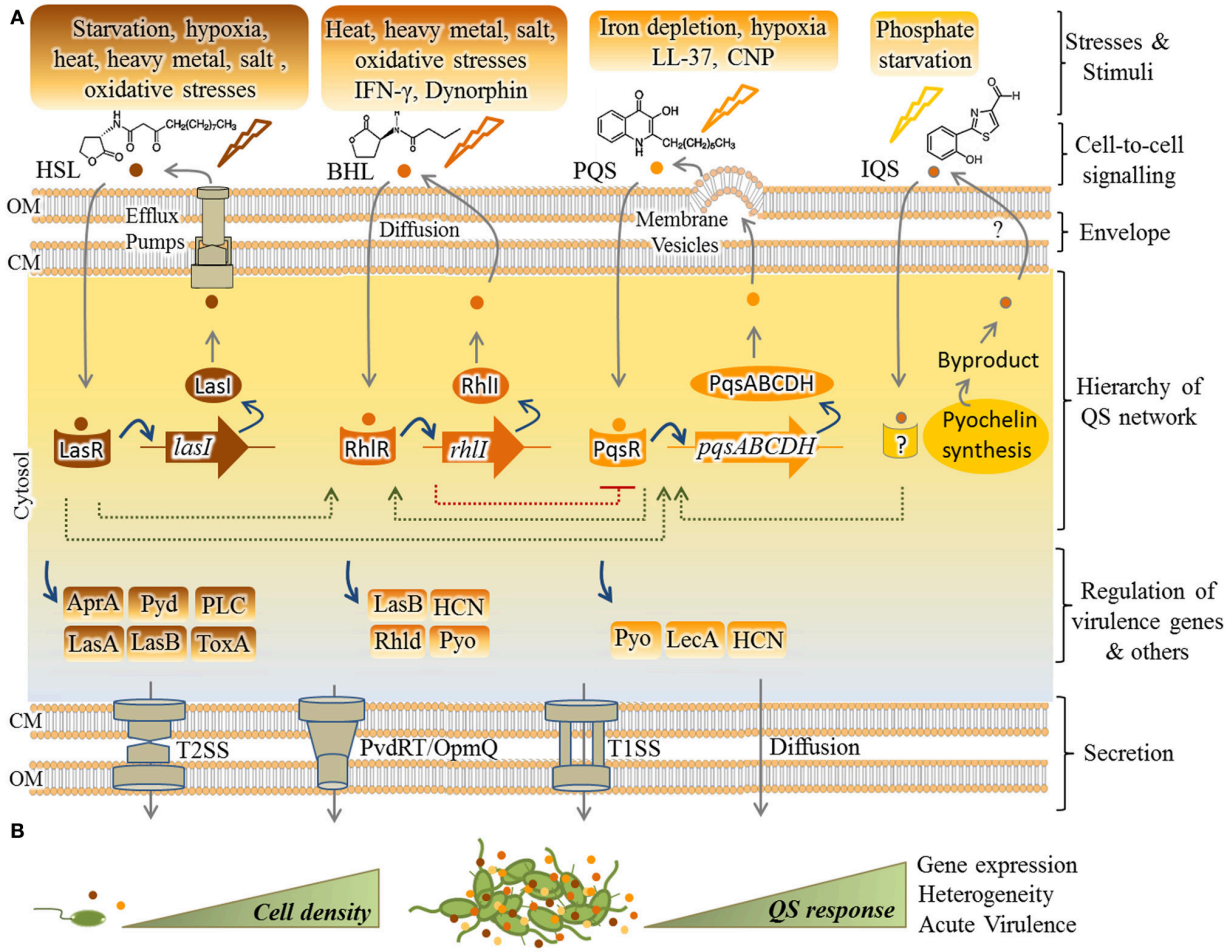
**Hierarchical QS network in *P. aeruginosa* and regulation of virulence factors. (A)** So far, four pathways including Las, Rhl, Pqs, and IQS have been understood as mediating QS responses in *P. aeruginosa* while LasR resides at the top of the cascade. In response to specific stimuli/stress, each pathway synthesizes cognate autoinducers (AIs) [HSL(3-oxo-C12-homoserine lactone)], BHL (*N*-butyrylhomoserine lactone or C4-HSL), PQS (2-heptyl-3-hydroxy-4-quinolone) and IQS [2-(2-hydroxyphenyl)-thiazole-4-carbaldehyde (aeruginaldehyde)]. Export and import of HSL, BHL, and PQS is mediated by the efflux pumps MexAB-OprM/MexEF-OprN, free diffusion and membrane vesicles, respectively. Question mark indicates unknown pathway of IQS transportation. As fine-tuned individual circuits, but interconnected (dashed lines), transcriptional factors (i.e., LasR, RhlR, and PqsR) are activated by AIs for upregulating expression of cognate AI synthases (respectively, LasI, RhlI, PqsABCDH) as well as others such as virulence factor genes. The IQS pathway remains unraveled and the IQS receptor is still unknown. Various secretion systems mainly type 1 and 2 secretion systems (T1SS/T2SS) and also PvdRT-OpmQ efflux pump mediate the secretion of virulence factors. **(B)** QS initiates upon cumulative production of AIs (small colorful circles) by increasing cell density and results in collective responses. AprA, alkaline protease; Pyd, pyoverdine; PLC, phospholipase C; Tox, toxin A; LasA, LasA elastase; LasB, LasB elastase; HCN, hydrogen cyanide; Pyo, pyocyanin; Rhld, rhamnolipids; Lec A, lectin A; CM, cytoplasmic membrane; OM, outer membrane.

As shown in Figure 1, four main pathways of QS dependent signaling exist in *P. aeruginosa*. These constitute a hierarchal network mediating integration of multiple signals via cross-talk between the QS signaling pathways. The most recently discovered IQS signaling pathway is less understood and its integration and impact on gene expression still needs to be unraveled. It was previously proposed that the IQS molecule (an aeruginaldehyde) is the product of enzymatic activity of proteins encoded by *ambBCDE* genes (Lee et al., 2013), while new findings showed the IQS molecule is a byproduct of the pyochelin biosynthesis pathway and AmbBCDE proteins are responsible for the biosynthesis of the toxin L-2-amino-4-methoxy-trans-3-butenoic acid (AMB) (Ye et al., 2014; Rojas Murcia et al., 2015; Sun et al., 2016).

In regard to the other three QS pathways, each system consists of at least two major functional elements; the first category of proteins (i.e., LasR, RhlR, or PqsR, respectively) is activated upon sensing specific autoinducers (AIs) and acts as transcriptional activator for genes encoding the second tier proteins, the cognate AI synthases (i.e., LasI, RhlI, and PqsABCDH, respectively). These activation steps constitute a fine-tuned circuit by which the synthesized AIs are exported outside the cells followed by being imported again (Figure 1). Transportation of these signals is not well understood, but it is proposed as being mediated by free diffusion, membrane transporters such as specific efflux systems or membrane vesicles (Mashburn and Whiteley, 2005; Martinez et al., 2009; Alcalde-Rico et al., 2016). Beyond this regulatory circuit the activated AI-sensing proteins act as transcriptional factors for activating the expression of other set of genes such as virulence genes in response to environmental stimuli (Figure 1). Transcriptional activation occurs via binding to conserved *las/rhl* boxes acting as operators residing upstream of these genes (González-Valdez et al., 2014; Lee and Zhang, 2015; Banerjee and Ray, 2016; Papenfort and Bassler, 2016). In the hierarchy of this network, LasR resides at the top of the cascade and along with RhlR mediates QS signaling at early stages of exponential growth phase while the PQS signaling pathway is active at late exponential growth phase (Choi et al., 2011). As abovementioned, cumulative and cell density-dependent production of AIs is required for reaching a specific threshold triggering collective responses by individual cells.

### QS-controlled virulence factors and stress responses

Production of virulence factors is a survival strategy for pathogens to evade the host immune defense resulting in progression of pathogenesis particularly at early stage of colonization and acute infection. A large number of virulence factors including cell-associated or secreted compounds, both low and high molecular weight compounds have been reported as important in colonization and establishment of infections by *P. aeruginosa*. Although they play a critical role in promoting bacterial growth and survival, they can cause devastating injuries to the host tissues and impair the immune responses. QS deficient mutants cause considerably less tissue damage and pathological changes during infections due to a significant decrease in the virulence and cytotoxicity (Nelson et al., 2009; Feng et al., 2016).

Production of many virulence factors is metabolically costly and requires community involvement. Hence, they are mainly under the regulatory control of the QS systems (Figure 1, Table 1; Whiteley et al., 1999; Diggle et al., 2002; Wagner et al., 2003; García-Contreras, 2016).

**Table 1:** **Key QS-dependent virulence factors produced by *P. aeruginosa***.

**Virulence factor**	**Class/Chemistry**	**Synthesis**	**Secretion**	**Property**	**Role in pathogenesis**	**References**
Pyocyanin	Secondary metabolite/ tricyclic phenazine	*phzA1-G1* & *phzA2-G2* operons	T2SS	Redox-active, zwitterion	Cytotoxic/damaging host cells, tissues and immune system cells/inducing apoptosis/ causing oxidative stress by mediating ${\text{O}}_{2}^{-}$ and H_2_O_2_ production	Britigan et al., 1992; Li et al., 2011; van 't Wout et al., 2015; Hall et al., 2016
Pyoverdine	Pyoverdines/ dihydroquinoline-type chromophore linked to a peptide	Large multimodular enzymes/ non-ribosomal peptide synthetases (NRPSs)	PvdRT-opmQ Efflux pump & MexAB-OprM efflux pumps	High affinity to Fe(III)/ iron acquisition/ fluorescent	Carrier of iron and other metals /crucial for infection and biofilm development	Visca et al., 2007; Hannauer et al., 2012; Schalk and Guillon, 2013
LasA Elastase	β-lytic zinc metallo-endopeptidases (staphylolytic)/ serine protease	*lasA*	The Sec pathway & T2SS	Protease and elastolytic activity/ cleaving a wide range of glycine-containing proteins	Staphylolytic activity/ enhancing the activity of LasB and host elastolytic proteases/ crucial for tissue invasion and infection	Toder et al., 1994; Kessler et al., 1997; Hoge et al., 2010
LasB Elastase	M4 thermolysin peptidase family/ zinc metalloprotease	*lasB*	The Sec pathway & T2SS	Protease and elastolytic activity	Degrading host proteins (e.g., elastin, collagen and fibrin)/ damaging host tissues/ inactivating key components of the immune systems/ Corneal amage/crucial for tissue invasion and infection	Toder et al., 1994; Hoge et al., 2010
Alkaline Protease (aeruginolysin)	M10 peptidase family/zinc- metalloendopeptid-ase	*aprA*	T1SS	Wide protease activity	Degrading tissue proteins such as laminin/destroying basal lamina/ causing hemorrhagic tissue necrosis/ inactivating key components of the immune systems/ crucial for tissue invasion	Guzzo et al., 1991; Hoge et al., 2010; Laarman et al., 2012
Lectin A	Tetrameric protein	*lecA* (or *pa1L*)	Intracellular; only a small fraction present on the cell surface	Galactophilic/ adhesive	Cytotoxic/impairing respiratory of epithelial cells/ inducing a permeability defect in the intestinal epithelium boosting ExoA penetration/ important for cell attachment, cell-cell interaction and biofilm development	Glick and Garber, 1983; Diggle et al., 2006; Chemani et al., 2009
PlcB	Phospholipases C	*plcB*	The Sec pathway & T2SS	Hydrolysing phosphatidylch-oline & phosphatidyleth-anolamine	Cytolytic activity/ important for cell membrane destruction and tissue invasion	Schmiel and Miller, 1999; Barker et al., 2004
Rhamnolipids	Rhamnose-containing glycolipidic compounds	*rhlAB* operon & *rhlC*		Biosurfactant/ detergent-like structure/ hemolytic activity	Cytotoxic/Causing tissue invasion and damage/ eliminating Polymorphonuclear neutrophilic leukocytes/ inhibiting the mucociliary transport and ciliary function of human respiratory epithelium/ important for maintenance of biofilm architecture and bacterial motility	Van Delden and Iglewski, 1998; Davey et al., 2003; Zhu and Rock, 2008; Alhede et al., 2009; Wittgens et al., 2011
Exotoxin A (ToxA)	PE belongs to the two-component AB toxin family/ NAD^+^-diphthamide-ADP-ribosyltransferase	*toxA*	T2SS	Modifying the elongation factor-2 in eukaryotic cells	A systemic and one of the most toxic virulence factor/cytotoxic by entering host cells/ inhibiting host protein synthesis/ causing cell death, tissue damage, bacterial invasion and immunosuppression/ crucial for keratitis	Pillar and Hobden, 2002; Daddaoua et al., 2012; Michalska and Wolf, 2015
Hydrogen cyanide (HCN)	Secondary metabolite	*hcnABC* operon	Diffusible	Highly toxic/ potent inhibitor of cytochrome c oxidase and other metalloenzymes	Cytotoxic/Suppressing aerobic respiration by rapid diffusion through tissues	Castric, 1975; Blumer and Haas, 2000; Lenney and Gilchrist, 2011; García-García et al., 2016

Analysis of bronchial secretions of CF patients during different stages of pulmonary exacerbations showed that QS upregulates the expression of genes involved in the production of some destructive virulence factors such as proteases (elastase, alkaline protease), phenazines (pyocyanin), toxins (exotoxin A), rhamnolipids and hydrogen cyanide (Jaffar-Bandjee et al., 1995; Lee and Zhang, 2015). Production of these toxic compounds is destructive to the host cells/tissues by impairing permeability barrier and by inhibiting protein production promoting cell death.

Recent findings suggested a correlation between systemic concentrations of some QS signaling molecules with the clinical status of pulmonary exacerbation and at least some QS signaling molecules were elevated at the start of either pulmonary exacerbation or antibiotic treatment when assessing different biofluids (Barr et al., 2015). In conclusion, virulence factors assist bacteria in colonization and survival aligned with worsened clinical course of infections. Thus, QS can determine the degree of pathological damages and clinical stages of infections in response to environmental factors.

Pathogenesis encompasses various stresses such as host immune factors, bacterial interspecies competition, phosphate/iron depletion and starvation. QS systems and production of some virulence factors mediate appropriate responses to these stresses to promote survival and adaptation (García-Contreras et al., 2015). Here, we provide some examples of stress responses mediated by the QS systems.

Interferon-γ (IFN-γ) is a crucial cytokine of the human immune system during infection and it coordinates a wide array of immunological responses such as up-regulation of pathogen recognition and the activation of bactericidal effector functions (Schroder et al., 2004). IFN-γ produced by T-cells was shown to bind directly to *P. aeruginosa* OprF, an outer membrane protein. Upon formation of IFN-γ-OprF complexes the *rhl* QS system was activated and resulted in up-regulation of the expression of *lecA* (or PA-I lectin) and synthesis of pyocyanin. The *lecA* gene encodes the virulence determinant, galactophilic lectin (or LecA) (Wu et al., 2005) which is cytotoxic and acts as adhesion factor mediating initiation of host recognition by *P. aeruginosa* (Chemani et al., 2009). It induces an increased permeability of the intestinal and respiratory epithelial cells enabling cytotoxic exoproducts such as exotoxin A (Laughlin et al., 2000) to enter host cells (Bajolet-Laudinat et al., 1994). In addition it also contributes to biofilm development (Diggle et al., 2006). Furthermore, the QS system has been reported to mediate a response to the host antimicrobial factor LL-37 by increasing the production of pyocyanin, hydrogen cyanide, elastase and rhamnolipids (Strempel et al., 2013). The QS-dependent production of rhamnolipids has a crucial role in neutralizing the attack of neutrophils due to their necrotic property (Jensen et al., 2007; Van Gennip et al., 2009).

Recent findings indicated that the LasR and RhlR QS systems, but not the PQS system, play major roles in adaptation and response to environmental stresses such as oxidative, heat, heavy metal and salt stresses (García-Contreras et al., 2015).

The stress response of *P. aeruginosa* to the depletion of phosphate and iron was found to be linked (Slater et al., 2003). Different studies showed that acquisition of phosphate and iron are important for survival and pathogenesis of *P. aeruginosa* and the expression of cognate genes mediating acquisition of these elements are upregulated upon interaction with human respiratory epithelial cells (Frisk et al., 2004; Chugani and Greenberg, 2007). Various studies unraveled that phosphate- and iron-deficient conditions can trigger the activation of the QS system especially via the IQS- or PQS-dependent pathway leading to boosted activation of central QS and the production of virulence factors such as rhamnolipids, phenazines, cyanide, exotoxin A, LasA protease, elastase, and antimicrobials (Kim et al., 2003; Long et al., 2008; Zaborin et al., 2009; Bains et al., 2012; Lee et al., 2013; Nguyen et al., 2015). Production of such virulence factors can increase cytotoxic impact of bacteria on host tissue and promote pathogen survival.

The recently discovered IQS system which controls the expression of a large set of virulence factors was shown to be directly activated by phosphate limitation in *P. aeruginosa* (Lee et al., 2013; Lee and Zhang, 2015).

*P. aeruginosa* QS signaling molecules such as 2-heptyl-4-hydroxyquinoline (HHQ) and 2-heptyl-4-hydroxyquinoline-*N*-oxide (HQNO) can serve as antimicrobial agents against *Staphylococcus aureus* which is commonly present during early stages of pulmonary infections in CF patients. It is proposed that this antibacterial activity supports the dominance of *P. aeruginosa* during the course of infection. Interestingly, this inter-species competition is linked to the availability of iron as the depletion of iron potentiates the antistaphylococcal activity of these metabolites (Nguyen et al., 2016). Also, LasA is a staphylolytic protease produced by *P. aeruginosa* and it is under the regulation of the *las* QS system (Toder et al., 1991). Furthermore, when *P. aeruginosa* was grown together with the yeast *Candida albicans* in a mixed biofilm, the QS system upregulated the production of virulence factors such as pyoverdine, rhamnolipids and pyocyanin (Trejo-Hernández et al., 2014).

Oxygen depletion known as hypoxia is another stress factor for *P. aeruginosa* during pathogenesis. Hypoxia condition is influenced by various factors such as reduced ventilation through viscose layers, chronic inflammation, microbial population and biofilm formation (Hassett, 1996; Worlitzsch et al., 2002; Yoon et al., 2002; Alvarez-Ortega and Harwood, 2007; Hassett et al., 2009). However, *P. aeruginosa* can survive and grow under hypoxia to high cell densities. Under hypoxia stress *P. aeruginosa* retains the capability of microaerobic respiration, although occurrence of nitrate respiration was thought to be possible (Alvarez-Ortega and Harwood, 2007). The QS regulon expression occurs at low oxygen conditions. Hammond et al. (2015) unraveled that the 4-hydroxy-2-alkylquinolines (HAQ)-dependent QS pathway is active during hypoxia via the ANR protein as the master transcriptional regulator of anaerobic respiration while it is in the absence of LasR signaling (Hammond et al., 2015). Under low oxygen tension, the ANR protein positively regulated the production of the QS signaling molecule 4-hydroxy-2-alkylquinolines and in turn the regulation of virulence-related genes could continue via PQS system (Hammond et al., 2015). Furthermore, under hypoxia stress the ANR protein and the QS systems cooperatively regulate hydrogen cyanide biosynthesis (Castric, 1983, 1994; Pessi and Haas, 2000). This study provided further evidence that low oxygen-dependent QS inversely correlates with denitrification i.e., suppresses nitrate respiration (Hammond et al., 2015).

Overall, these examples have provided further insight into the versatility of *P. aeruginosa* to adapt to various environmental conditions by processing signals via integrated and cross-talking QS pathways resulting in enhanced survival i.e., in a medical context establishment of acute and chronic infections.

## Persistence and biofilm formation

During acute infection the relationship between pathogen and host is reciprocally devastating as a variety of cytotoxic molecules produced by bacteria impair the host cellular processes while bacteria on the other hand encounter immune system responses such as production of antimicrobial compounds and reactive oxygen species, as well as enhanced phagocytosis. In this context, motile *P. aeruginosa* display a more virulent phenotype. Various modes of *P. aeruginosa* motility such as swimming and swarming involving flagella and twitching using type 4 pili are associated with virulent traits (Winstanley et al., 2016). A motile cell is readily detectable by the host immune system via flagellar and/or other motility components mediating recognition and induction of signaling pathways which trigger inflammatory responses and phagocytosis by murine or human macrophages (Amiel et al., 2010).

Switching to sessile lifestyle along with lower virulency is a survival advantage by which many pathogenic bacteria such as *P. aeruginosa* evade stresses and adverse conditions. They lose motility and attach to surfaces and form cellular aggregations or microcolonies which are embedded in extracellular polymeric substances (EPS) to protect bacteria from the surrounding environment. These structures are so called biofilms conferring an extreme capacity for persistence against phagocytosis, oxidative stresses, nutrient/oxygen restriction, metabolic waste accumulation, interspecies competitions, and conventional antimicrobial agents (Leid, 2009; Olsen, 2015).

Formation of mucoid biofilm by *P. aeruginosa* is the hallmark of chronic infections and indicative of disease progression and long-term persistence. As a consequence, *P. aeruginosa* dominates the microbial community of CF lungs in patients older than 24 years (McDaniel et al., 2015).

Other *P. aeruginosa* biofilm associated infections include chronic wound infection, chronic otitis media, chronic rhinosinusitis, catheter-associated urinary tract infection, and contact lens-related keratitis (Römling and Balsalobre, 2012).

### Composition of the *P. aeruginosa* biofilm

Formed on abiotic and biotic surfaces, the matrix of most biofilms embedding bacterial cells may account for over 90% of dry weight of whole biofilm mass. In fact, this matrix creates a niche rendering bacteria for intense cell-cell interaction and communication as well as a reservoir of metabolic substances, nutrients and energy for promoting growth while shielding cells from unfavorable conditions (Flemming and Wingender, 2010). The matrix is mainly formed by extracellular polymeric substances (EPS) which are mainly polysaccharides, proteins, extracellular DNA (eDNA) and lipids (Strempel et al., 2013). Major polymers and relevant characteristics are listed in Table 2.

**Table 2:** **Key polymeric substances in *P. aeruginosa* biofilm formation and development**.

**Name**	**Identity/Chemistry**	**Precursor(s)**	**Biosynthesis**	**Property**	**References**
Psl	Exopolysaccharide / Repeating pentasaccharide containing D-mannose, D-glucose and L-rhamnose	GDP-D-mannose, UDP-D-glucose and dTDP-L-rhamnose	*The pslA-O operon*	Neutral charge	Byrd et al., 2009; Colvin et al., 2012
Pel	Exopolysaccharide/ Partially acetylated (1 → 4) glycosidic linkages of N-acetylgalactosamine and N-acetylglucosamine.	UDP-sugar nucleotide/ uncharacterized	The *pelA-G* operon	Positively charged	Franklin et al., 2011; Jennings et al., 2015
Alginate	Exopolysaccharide/ O-acetylated 1–4 linked D-mannuronic acid and variable proportions of its 5-epimer L-guluronic acid	GDP-mannuronic acid	The alginate operon (*algD, alg8, alg44, algK, algE, algG, algX, algL, algI, algJ, algF*, and *algA) and algC*	Negatively charged	Hay et al., 2010; Moradali et al., 2015
eDNA	Nucleic acid	−	Cell lysis	Negatively charged	Allesen-Holm et al., 2006; Ma et al., 2009
Type 4^*^ pili	Multiprotein complex/ Type 4a pili	−	The *pilM/N/O/P/Q* and the *fimU-pilVWXY1Y2E* operons	−	Ayers et al., 2009; Burrows, 2012
Flagella^*^	Multiprotein complex	−	At least 41 genes clustered in three regions of the genome encode flagellin structural and regulatory components	−	Jyot and Ramphal, 2008

* *Are not commonly considered as classical matrix molecules of biofilm, but important for biofilm maturation*.

The exopolysaccharides Psl, Pel, and alginate are major constituents of the *P. aeruginosa* biofilm matrix involved in surface adhesion and together with eDNA determine the biofilm architecture. These EPS play an important role in resistance to immune responses and antibiotic treatments (Ghafoor et al., 2011; Gellatly and Hancock, 2013; Strempel et al., 2013). The differential role of each EPS has been analyzed at each stage of biofilm development. The various exopolysaccharides and eDNA were shown to interactively contribute to the biofilm architecture (Ghafoor et al., 2011). The presence of various EPS exhibiting different physiochemical properties confers a survival strategy for increasing the flexibility and stability of biofilms under various conditions (Jennings et al., 2015).

The Psl polysaccharide is a key element at early stage of biofilm formation when cells explore surfaces for adhesion (Overhage et al., 2005). It is anchored around cells in a helical arrangement initiating biofilm formation by enhancing cell migration, cell-cell interaction and cell-surface adhesion whereas in mature biofilms it is located to the periphery of mushroom shaped macrocolonies (Ma et al., 2009; Zhao et al., 2013). Psl can exist as a fiber-like matrix requiring type 4 pili-mediated migration of cells (Wang S. et al., 2013). It protects cells against phagocytosis and oxidative stress during infection (Mishra et al., 2012). Recent studies suggested that Psl can provide an instant protective role against anti-biofilm agents and a broad spectrum of antibiotics particularly at early stage of biofilm development (Zegans et al., 2012; Billings et al., 2013). Therefore, Psl provides a survival advantage during pathogenesis.

Similar to Psl, Pel is important for initiating and maintaining cell–cell interaction in biofilms (Colvin et al., 2011). Pel and/or Psl are the primary matrix structural polysaccharides in non-mucoid *P. aeruginosa* strains as a predominant environmental phenotype. However, contribution of Psl and Pel to the structure of mature biofilms is strain-dependent while both unique and functionally redundant roles have been reported for these exopolysaccharides (Colvin et al., 2012). Recent studies elucidating the chemical structure and biological function of Pel demonstrated that it is a major structural component of the biofilm stalk where it cross-links eDNA and structurally compensates for the absence of Psl in the periphery of mature biofilm (Jennings et al., 2015). Furthermore, Pel was shown to protect bacteria against certain aminoglycoside antibiotics (Colvin et al., 2011).

Overproduction of the exopolysaccharide, alginate, is characteristic for mucoid phenotype of most clinical isolates from CF patients. During adaptation to the CF lung environment, alginate is overproduced and predominantly constitutes the matrix of mature biofilms conferring a slimy or mucoid phenotype. Indeed, it is greatly important in biofilm maturation, structural stability and protection as well as persistence by shielding *P. aeruginosa* cells against opsonophagocytosis, free radicals released from immune cells, and antibiotics used for treatment (Hay et al., 2009a; Hay I. D. et al., 2013; Strempel et al., 2013). Some *in vitro* biofilm studies showed that the composition of the alginates can greatly influence biofilm characteristics such as viscoelastic property, bio-volume, cell density and architecture as well as cell-to-cell interaction, cell aggregation and surface attachment (Tielen et al., 2005; Moradali et al., 2015).

As abovementioned, eDNA is another important structural component for biofilm development and along with the Pel polysaccharide it can be detected within the stalks of mushroom-shaped macrocolonies. However, eDNA has multifaceted roles in biofilm formation such as contribution to forming cation gradients in the matrix via the chelating interaction of highly anionic DNA with cations such as Mg^2+^, Ca^2+^, Mn^2+^, and Zn^2+^, as a nutrient source during starvation, facilitating twitching motility and coordinating cell movements and conferring antibiotic resistance (Allesen-Holm et al., 2006; Mulcahy et al., 2008; Gloag et al., 2013).

Among the proteinaceous biofilm constituents, both flagella and the type 4 pili are important during maturation of the biofilm, however, these cell appendages are not commonly considered as classical matrix components of biofilms. Type 4 pili are important for adhesion and promote initial attachment of cells to surfaces at early stage of biofilm formation. Together with eDNA, flagella and the type 4 pili mediate migration required for the formation of the stalk and the cap in the mushroom-shaped microcolonies of the mature biofilm (Table 2; Barken et al., 2008; Mann and Wozniak, 2012).

### Central regulatory network governing the motility-sessility switch

**Figure 2:**
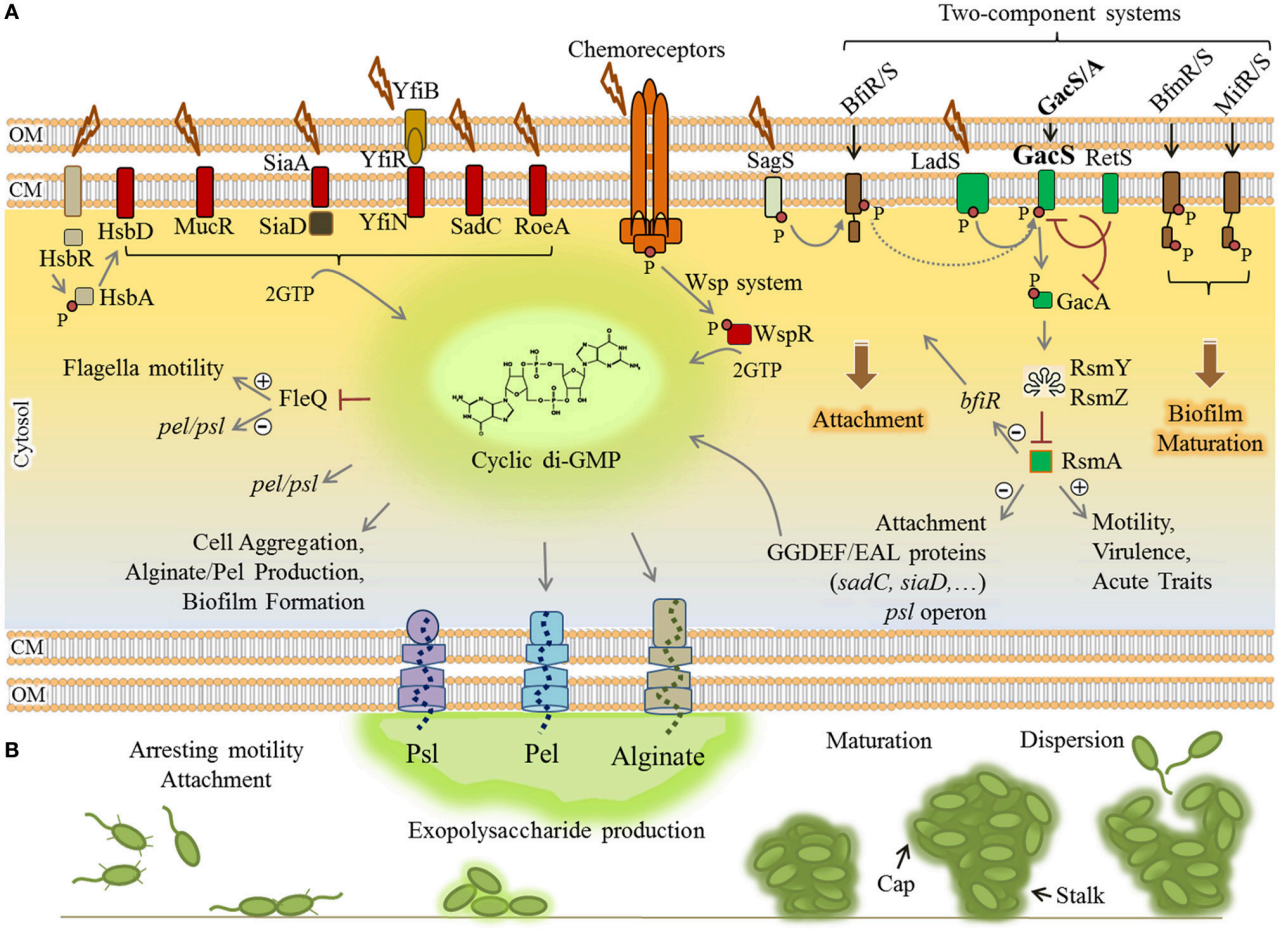
**Regulatory networks underlying biofilm formation by *P. aeruginosa*. (A)** Elevation of the cyclic di-GMP molecule is a key determinant for the motility-sessility switch. Environmental cues are sensed by various proteins localized in the envelope of the cells where these proteins contribute to two-component systems (brown/green rectangles), chemoreceptor-like system (orange complex) and other receptor mediated signaling pathways (arranged in the left side of figure). Either triggered as phosphorylation cascades (small red circle) or protein-protein interactions, the signals induce diguanylate cyclases (containing GGDEF motif) (red rectangles) to synthesize cyclic di-GMP from two molecules of GTP (guanosine-5′-triphosphate). Consequently, cyclic di-GMP sensing proteins act as receptor/effector for specific outputs such as induction of alginate and Pel polymerization, inhibition of motility and derepression of *psl/pel* expression via FleQ, induction of attachment and biofilm formation/maturation triggered by two component systems. The two-component systems are interconnected and the LadS/RetS/GacS/GacA/RsmA regulatory network (green rectangles) plays a key role in the phenotypic switch from motility to sessility and downregulation of QS and virulence factor production. **(B)** Various stages of biofilm formation and development were represented. Plus and minus signs represent positive and negative effect of transcriptional regulators, respectively. CM, cytoplasmic membrane; OM, outer membrane.

Transition from motility to sessility requires dynamic regulatory networks at transcriptional, post-transcriptional and post-translational levels resulting in coordinated timely expression of hundreds of genes. These events mainly arrest flagella based motility and the production of virulence factors such as exotoxins and proteases while positively regulating surface attachment, EPS production and biofilm maturation (Figure 2).

The small molecule cyclic-3′5′-diguanylic acid (cyclic di-GMP) is a key signal in post-transcriptional regulation of biofilm formation. It is an almost ubiquitous second messenger present in a wide range of bacteria that principally controls motility-sessility switch. The major determinant for this substantial phenotypic change is the cellular level of cyclic di-GMP, so that its elevation triggers biofilm formation while inhibiting motility. The cyclic di-GMP signaling system is very complex and two groups of proteins have been identified as main actors. The first group comprises cyclic di-GMP metabolizing enzymes including diguanylate cyclases (DGC) (containing GGDEF motif) and phosphodiesterases (containing EAL or HD-GYP motif) that respectively synthesize and degrade cyclic di-GMP in cells (Römling et al., 2013; Valentini and Filloux, 2016). At least 40 such proteins directly synthesize and/or degrade cyclic di-GMP in *P. aeruginosa* which controls cellular level of this molecule in response to perceived stimuli (Ryan et al., 2006). The second group is represented by cyclic di-GMP sensing proteins which also act directly as effectors or via protein-protein interactions to mediate the output response (Römling et al., 2013). For example, cyclic di-GMP is essential for the activation of alginate polysaccharide polymerization (Remminghorst and Rehm, 2006). Experimental evidence indicated that a pool of cyclic di-GMP is synthesized by MucR (a hybrid GGDEF/EAL domain-containing protein) in the proximity of the alginate biosynthesis/secretion multi-protein complex of *P. aeruginosa* (Hay et al., 2009b; Wang et al., 2015). Cyclic di-GMP binds to PilZ domain of Alg44 protein and allosterically activates alginate polymerization via interaction with Alg8 glycosyltransferase (Hay et al., 2009b; Moradali et al., 2015; Wang et al., 2015). Also, cyclic di-GMP binding to FleQ, a transcriptional master regulator represses flagella biosynthesis while it concomitantly derepresses the expression of *pel* and *psl* genes (Baraquet et al., 2012; Figure 2). Likewise, there are many other receptor/effector proteins which enhance required pathways for biofilm formation upon cyclic di-GMP binding while they inhibit motility and other virulence factor synthesis pathways.

In addition, the Wsp chemosensory system in *P. aeruginosa* is homologous to chemotaxis signaling pathways which regulate cyclic di-GMP synthesis via signal transduction (Figure 2). A cascade of phosphorylations is triggered upon surface attachment and possibly sensing mechanical stress or other environmental stimuli which then activate the cyclic di-GMP synthesizing protein WspR promoting biofilm formation (Hickman et al., 2005; Porter et al., 2011).

Furthermore, transduction of phosphorylation events via two-component regulatory systems controls biofilm formation in a stage-specific manner (Figure 2). This network consists of BfiRS, BfmRS, and MifRS and GacS/GacA regulatory components (Petrova and Sauer, 2009). The GacS/GacA two-component system is part of the global regulatory pathway comprising LadS/RetS/GacS/GacA/RsmA proteins (Figure 2). This pathway controls many physiological responses at post-transcriptional level and is involved in both motility-sessility and acute-chronic infection transitions. Of this regulatory pathway, the RNA-binding protein RsmA negatively controls biofilm formation pathways while it induces production of T3SS, type 4 pili and other virulence factors. RsmA binds to *psl* mRNA and inhibits the translation of required proteins for Psl polysaccharide biosynthesis (Irie et al., 2010; Jimenez et al., 2012). It also represses production of GGDEF/EAL encoding proteins; hence, it inhibits elevation of cyclic di-GMP levels. In *P. aeruginosa*, under stress conditions, this pathway generates non-coding RNAs (ncRNAs) known as RsmY and RsmZ which counteract RsmA translational repression activity, consequently derepressing biofilm formation mainly via cyclic di-GMP level increase resulting in exopolysaccharides production (Jimenez et al., 2012).

There are other regulatory pathways known to be involved in cyclic di-GMP turnover in response to external stimuli, but the further precise function still remains to be elucidated (Figure 2).

### The role of QS in biofilm development and maturation

In addition to abovementioned regulatory networks, biofilm residents utilize QS systems for cell-to-cell communication and spatio-temporal regulation of expression of specific genes. During chronic infection, a major proportion of the colonizing population was thought to lose QS due to hypermutation events and phenotypic alterations. However, further investigations have now revealed that genes involved in the progress of biofilm maturation and persistence are positively regulated by QS in *P. aeruginosa*. Indeed QS-deficient mutants of *P. aeruginosa* (i.e., Δ*lasR*Δ*rhlR* and Δ*lasI*Δ*rhlI*) formed thin and much less developed biofilms which were more sensitive to antibiotic treatments and eradication (Shih and Huang, 2002; Nelson et al., 2009). Furthermore, Bjarnsholt et al. (2010) demonstrated that at least a part of QS pathways i.e., *rhl* encoded system and the production of C4-HSL signals was retained in predominantly mucoid population at the end of chronic stages coinciding with overproduction of alginate and rhamnolipids (Bjarnsholt et al., 2010). The biosurfactants, rhamnolipids, have been suggested to play an active role in maintenance of the biofilm architecture by contributing to the formation of internal cavities within the mature biofilm, allowing proper flow of water and nutrients (Davey et al., 2003; Boles et al., 2005; Dusane et al., 2010; Chrzanowski et al., 2012). Additionally, the production of pel polysaccharide, eDNA and QS-controlled production of pyocyanin are critical for biofilm maturation. Pel cross-links eDNA in the biofilm stalk via ionic interactions and it serves as important structural components of the biofilm matrix of *P. aeruginosa* (Jennings et al., 2015). Furthermore, pyocyanin molecules can promote eDNA release by inducing bacterial cell lysis. Pyocyanin binds to eDNA increasing its solution viscosity which influences the physicochemical interactions of the biofilm matrix with environment as well as facilitates cellular aggregations (Das et al., 2013, 2015). Collectively, such molecular and cellular interactions in combination with other polymeric substances lead to establishment of a robust and mature biofilm.

## Antibiotic resistance mechanisms

Indeed, the emergence of antibiotic resistant bacteria is a global health issue. Among identified notoriously multi-drug resistant (MDR) bacteria, *P. aeruginosa* has been introduced as a major concern with a growing threat to global health resulting in dramatically increasing prevalence of nosocomial and chronic infections. This is due to the extraordinary capacity of these bacteria to develop resistance against a wide range of antimicrobials through various molecular mechanisms which are often simultaneously present in clinical isolates. Although each resistance mechanism is related to a specific class of antibiotics, multiple mechanisms mediate variably resistance to each class of antibiotics (Potron et al., 2015). Furthermore, the contribution of each mechanism varies from country to country. Loss or reduced copy numbers of OprD and overproduction of active efflux pumps, AmpC β-lactamase and extended-spectrum β-lactamases have been mainly reported as main contributors to multi-drug resistance phenotypes of *P. aeruginosa* isolates.

Recent reviews have described the prevalence and contribution of each resistance mechanism to each class of antibiotics in detail (Lister et al., 2009; Strateva and Yordanov, 2009; Sun et al., 2014; Hong et al., 2015; Potron et al., 2015). Here, we reviewed the most frequent and well-understood findings which are classified into intrinsic, acquired and adaptive mechanisms, and we provide an update on our understanding of how *P. aeruginosa* can survive antibiotic treatments.

### Intrinsic resistance mechanisms

**Figure 3:**
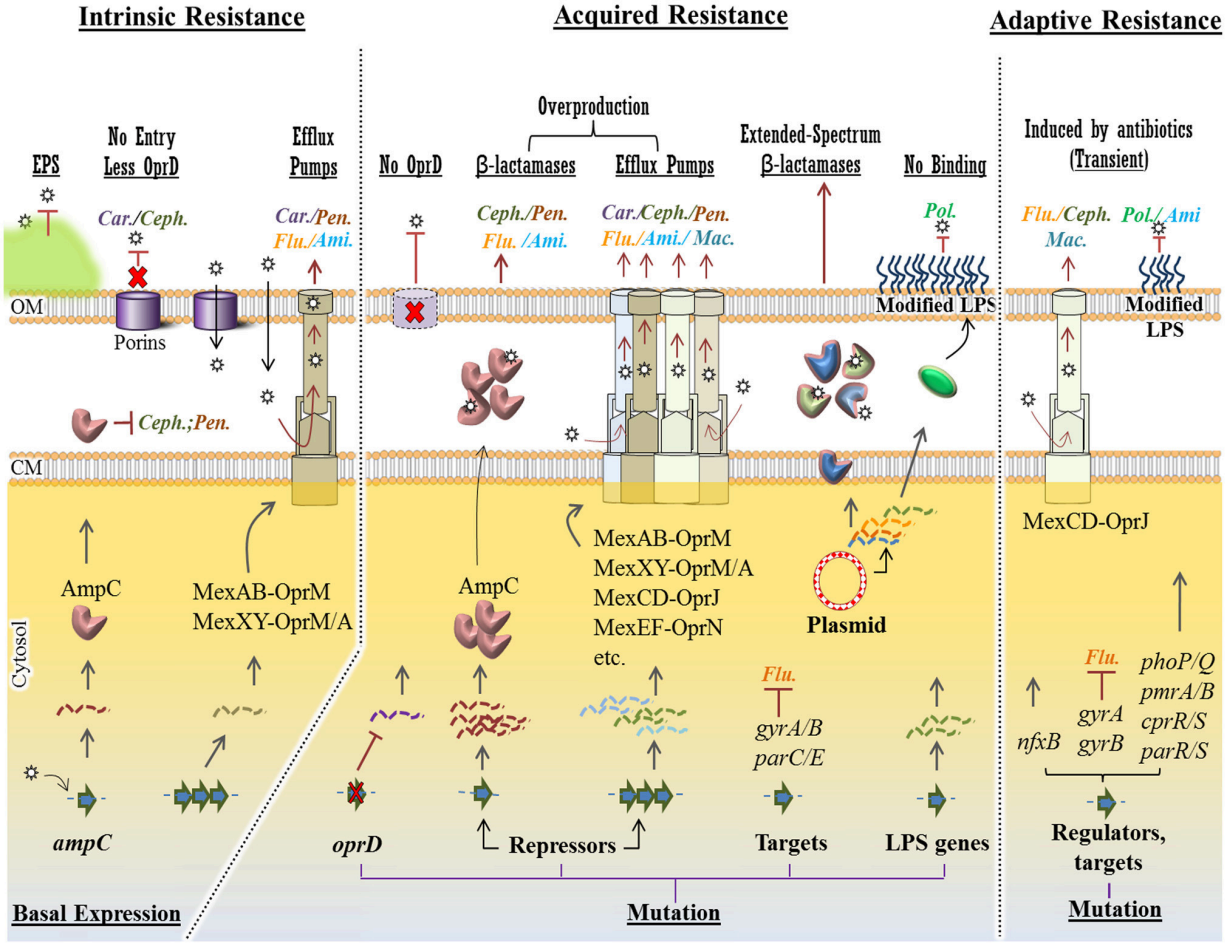
**Intrinsic, acquired and adaptive mechanisms confer antibiotic resistance in *P. aeruginosa***. For each mechanism, various molecular strategies, which confer resistance to specific class of antipseudomonal antibiotics (Car., Carbapenems; Ceph., Cephalosporins; Pen., Penicillins; Ami., Aminoglycosides; Flu., Fluoroquinolones; Mac., Macrolides and Pol., Polymyxins), were presented at the top of the figure (underlined) Intrinsic mechanisms such as structural barriers [e.g., EPS (extracellular polymeric substances)], OprD reduction and basal production of AmpC β-lactamase and MexAB/XY efflux pumps confer a basal resistance to some group of antibiotics. However, in acquired resistance, mutational changes in the *oprD* gene, transcriptional repressors causingupregulation of resistance genes and efflux pumps conferring resistance against a wider spectrum of antibiotics. Plasmid-mediated resistance is very potent as a variety of resistance genes can be exchanged among bacteria. Either mediated by mutational changes in the genome or in plasmids, resistance to polymyxins occurs via modification of LPS (lipopolysaccharide) components hindering binding of the antibiotic to this layer. Adaptive resistance occurs in the presence of antibiotics mainly via mutation in regulatory genes. This is a transient and reversible resistance, which will reverse upon removal of antibiotics. Stars represent antibiotics and dashed/wavy lines represent transcriptional levels of each gene product. CM, cytoplasmic membrane; OM, outer membrane.

Like many Gram-negative bacteria, *P. aeruginosa* can be intrinsically resistant to particular antibiotics. Such intrinsic resistance mechanisms stem from the existence of genes in bacterial genome encoding inherent properties of cell structures and composition providing protection against toxic molecules and antimicrobials. It can also be contributed by the lack of susceptible sites which naturally exist in antibiotic sensitive species (e.g., resistance to triclosan) (Lambert et al., 2011; Blair et al., 2015; Figure 3).

However, hydrophilic antibiotics can enter cells by diffusing through membrane channels or porin proteins in a non-specific manner. As one of the intrinsic mechanisms, *P. aeruginosa* limits antibiotic entry by reducing the number of non-specific porin proteins and replacing them with specific or more-selective channels for taking up required nutrients resulting in lowered permeability to toxic chemicals (Tamber and Hancock, 2003; Figure 3). *P. aeruginosa* resistance to currently used broad-spectrum drugs such as carbapenems and cephalosporins is commonly caused by this adaptation (El Amin et al., 2005; Baumgart et al., 2010). Many of the clinical strains of *P. aeruginosa* displaying resistance to carbapenems such as imipenem are deficient in the OprD porin which specifically facilitates the diffusion of basic amino acids, small peptides as well as carbapenems into the cell (Trias and Nikaido, 1990; Strateva and Yordanov, 2009).

Active multidrug efflux pumps greatly contribute to antibiotic resistance observed in *P. aeruginosa*. The involved genes are ubiquitous in Gram-negative bacteria and they are located on the genome or plasmids. The multidrug efflux pumps are multi-protein complexes spanning the envelope of Gram-negative bacteria. They are responsible for expelling various toxic materials and a wide range of antimicrobials. Because of their broad substrate specificities they display resistance against different classes of antibiotics which are chemically unrelated (Blair et al., 2015; Venter et al., 2015).

*P. aeruginosa* possesses four well known active multidrug efflux pumps including MexAB-OprM, MexXY/OprM(OprA), MexCD-OprJ, and MexEF-OprN (Figure 3). The gene sets encoding these systems are under different regulatory factors; therefore, the expression levels of these systems significantly differ under various conditions. The MexAB-OprM and MexXY/OprM(OprA) are the most clinically important sets due to their large prevalence in clinical strains and significant contribution to a wide range of antibiotics (Avrain et al., 2013). The *mexAB-oprM* genes show a stable and constitutive expression in the cell guaranteeing a protective basal level production of the MexAB-OprM system to consistently expel a wide range of toxic molecules and antibiotics (Li et al., 2015). Hence it mainly contributes to natural resistance to antibiotics. The *mexXY-(oprA)* genes show lower basal expression levels and are mainly induced in response to protein synthesis inhibitors that target the ribosomal machinery (Matsuo et al., 2004; Hay T. et al., 2013). Both *mexCD-oprJ* and *mexEF-oprN* genes are not typically expressed in wild-type strains or their expression is very low and they have been proposed not to contribute significantly to natural antibiotic resistance (Llanes et al., 2011; Li et al., 2015).

There are other forms of multidrug efflux pumps such as MexJK, MexGHI-OpmD, MexVW, MexPQ-OpmE, MexMN, and TriABC. They are not expressed in wild-type strains but may contribute to adaptive resistance against antibiotic or biocide agents when expressed in resistant strains (Lister et al., 2009; Avrain et al., 2013).

On the other hand, they might play role in other physiological pathways as well. For example, The MexEF-OprN and MexGHI-OpmD sets can modulate QS systems by exporting the quinolone signaling molecule PQS reducing its cellular concentration resulting in the reduction of virulence factor production, which is presumably in favor of establishment of chronic infections (Köhler et al., 2001; Aendekerk et al., 2005; Lamarche and Déziel, 2011). However, many of these mechanisms remain still unclear with regard to their connection with other physiological pathways and their clinical relevance.

Another player of intrinsic resistance and basal lower level antibiotic susceptibility in *P. aeruginosa* is the gene encoding an inducible β-lactamase (AmpC) (Figure 3). Particularly, chromosomal expression and production of AmpC confers low level resistance to aminopenicillins and most cephalosporins because these antibiotics strongly induce the production of AmpC which consequently hydrolyzes these substrates (Oliver et al., 2015). However, through adaptive or acquired resistance mechanisms AmpC can be overproduced, consequently conferring resistance to a wider range of antibiotics such as aminoglycosides and fluoroquinolones (Umadevi et al., 2011). These mechanisms will be further discussed later.

### Acquired resistance mechanisms

*P. aeruginosa* can acquire resistance to antibiotics through mutation of intrinsic genes or horizontal acquisition from other bacteria through transferring plasmids carrying genetic materials encoding for antibiotic resistance (Davies, 1997; Davies and Davies, 2010). Contrary to intrinsic mechanisms, acquired resistance is related to antibiotic selection and this selective advantage occurs in the presence of antibiotic compounds leading to irreversible resistant population (Lee et al., 2016). Therefore, similar to intrinsic resistance, acquired resistance is stable too and it can be transmitted to progeny.

However, due to over-expression of resistance genes and transmissibility by plasmids, acquired resistance is a potent mechanism which confers resistance to a wide spectrum of antibiotics as well as leads to increased prevalence among clinical and environmental strains.

### Boosted antibiotic resistance via mutations

Intrinsic resistance genes are negatively or positively regulated by one or more regulatory mechanisms which confer a basal lower susceptibility of *P. aeruginosa* to a narrow spectrum of antibiotics. However, mutation in regulatory pathway could increase promoter activities resulting in unleashing gene expression and overproduction of protein products such as AmpC and multi-drug efflux pumps systems. Consequently, it causes higher level of resistance to antibiotics (Blair et al., 2015; Figure 3).

As a common mutational feature of *P. aeruginosa* isolates, resistant clinical mutants display a constitutive high level of AmpC production even in the absence of antibiotic inducers. This is mainly due to mutational inactivation of *ampD* (repressor of *ampC*) and specific point mutations of *ampR*, both encoding two regulatory proteins important in induction of the *ampC* gene (Juan et al., 2005; Figure 3). Consequently, it turns into a major cause of greater resistance to a wide range of antibiotics such as most of the β-lactams (e.g., ticarcillin and piperacillin) as well as monobactams, third-generation and fourth-generation cephalosporins (Lister et al., 2009; Berrazeg et al., 2015). One study showed that 73% of tested clinical strains showed AmpC overproduction (Henrichfreise et al., 2007).

Several regulatory loci such as *mexR, nalD, nalB*, and *nalC* negatively control the expression of the *mexAB*-*oprM* operon in *P. aeruginosa*. On the other hand, various loss-of-function mutations in these loci derepress the expression of the *mexAB*-*oprM* operon leading to the overproduction of MexAB-OprM complex conferring a greater resistance to carbapenem antibiotics (Quale et al., 2006; Lister et al., 2009; Kao et al., 2016; Figure 3). Likewise, overproduction of other multidrug efflux pumps such as MexXY and MexCD-OprJ can occur via mutations in regulatory loci leading to unleashing gene expression and a greater resistance to a variety of antimicrobial agents (Lister et al., 2009; Figure 3).

Another clinically important and prevalent mutational alteration is attributed to OprD porin channel. This porin channel is localized in the outer membrane of *P. aeruginosa* and it is characterized as a carbapenem-specific porin (Figure 3). Therefore, loss or reduction of OprD can reduce permeability of the outer membrane to carbapenems (Epp et al., 2001; Gutiérrez et al., 2007; Kao et al., 2016). The emergence of resistance to imipenem and reduced susceptibility to meropenem has been reported upon the occurrence of *oprD* mutations. Genetic alteration in *oprD* can occur via nucleotide insertion or deletion and point mutations resulting in frameshift of the gene sequence, amino acid substitution, shortened putative loop L7 and premature stop codons (Kao et al., 2016). Furthermore, downregulation of *oprD* expression can be mediated by other regulatory factors such as MexT which itself concurrently upregulates *mexEF*-*oprN* expression (Köhler et al., 1997; Ochs et al., 1999).

Additionally, fluoroquinolone resistance among *P. aeruginosa* isolates can be mediated by either mutational changes within the fluoroquinolone targets i.e., DNA gyrase (*gyrA* and *gyrB*) and/or topoisomerase IV (*parC* and *parE*) or overproduction of active or inducible efflux pumps (Lee et al., 2005; Sun et al., 2014; Figure 3).

### Plasmid-mediated resistance

Bacterial plasmids serve a central role as a potent vehicle for acquiring resistance genes and subsequent delivery to recipient host. This is so-called horizontal gene transfer whereby genetic elements can be transferred between bacterial cells particularly via conjugation. Some resistance plasmids are broad host range which can be transferred among various species via bacterial conjugation, while narrow host range plasmids are transferred among a small number of cells from similar bacterial species. For example, plasmid RP1 can transfer resistance genes to most Gram-negative bacteria (Kenward et al., 1978).

Plasmid-encoded antibiotic resistance confers resistance to different classes of antibiotics that are currently applied in frontline of clinical treatments such as β-lactams, fluoroquinolones and aminoglycosides (Bennett, 2008; Figure 3). So far, *P. aeruginosa* resistance via horizontal gene transfer has been reported for the genes encoding β-lactam-hydrolyzing enzymes known as the extended-spectrum β-lactamases and the carbapenemases, aminoglycoside-modifying enzymes, 16S rRNA methylases resulting in high-level pan-aminoglycoside resistance (Poole, 2011).

The genes encoding extended-spectrum β-lactamases and carbapenemase are clinically important not only due to their hydrolyzing activity on a wide range of β-lactams such as carbapenems and extended-spectrum cephalosporins, but also for their worldwide prevalence (Paterson and Bonomo, 2005; Blair et al., 2015; Sullivan et al., 2016). The global epidemiology of carbapenem-resistant *P. aeruginosa* was recently analyzed by Hong et al (Hong et al., 2015). They reported that the geographical prevalence of these genes differs from country to country, whereas the genes encoding carbapenemases such as IMP, VIM, and NDM type metallo-β-lactamases have been found in all continents (Johnson and Woodford, 2013; Meletis and Bagkeri, 2013; Hong et al., 2015). Almost all types of transferable carbapenemases, except SIM-1, have been detected in *P. aeruginosa*, and the prevalence of carbapenem-resistant isolates of *P. aeruginosa* is gradually increasing (Meletis and Bagkeri, 2013; Hong et al., 2015).

It is of concern that transferable plasmids carrying some of the resistance genes are mobile among a wide range of unrelated Gram-negative bacteria which increases the antimicrobial resistance transfer rate causing increasing treatment complications (Hong et al., 2015). Recent findings about antibiotic resistance have been even more concerning and warning. Liu et al. reported the first evidence of plasmid-mediated colistin resistance from China (Liu et al., 2016; Figure 3). Colistin (or polymyxin E) belongs to the family of polymyxins. The members of this class of antibiotics such as polymyxin B and colistin have been the last resort for antibiotic treatment of carbapenem-resistant bacteria such as *P. aeruginosa* isolates and Enterobacteriaceae (Falagas and Kasiakou, 2005). Resistance to polymyxins was previously reported to occur via chromosomal mutations (Moskowitz et al., 2012; Gutu et al., 2013), however, new evidence suggests plasmid-mediated resistance through the mobilization of the *mcr-1* gene which consequently confers resistance to colistin (Figure 3). This gene was discovered in *E. coli* strain SHP45 collected from agricultural products. It is more concerning that the plasmid carrying *mcr-1* was mobilized into *K. pneumoniae* and *P. aeruginosa* via conjugation (Liu et al., 2016). This finding has triggered serious concerns about the emergence of pan-drug-resistant Gram-negative bacteria leading to almost untreatable infections. Recent findings provided some evidence of the spreading high-risk of clone ST654 of *P. aeruginosa* containing the genomic *bla*_NDM−1_ resistance gene which also conferred resistance to colistin. It is likely that *bla*_NDM−1_ was acquired via genetic exchange between *P. aeruginosa* and *K. pneumoniae* isolate in the same patient (Mataseje et al., 2016).

### Adaptive resistance mechanisms

Compared to other types of resistance mechanisms, adaptive mechanisms are not really well understood. Adaptive resistance is an unstable and transient form of resistance, which is induced in the presence of specific antibiotics and other environmental stresses. This type of resistance mainly relies on induced alterations in gene expression and protein production or alterations in antibiotic targets and it is reversal upon removal of external stimuli leading to re-gaining susceptibility (Barclay et al., 1992; Xiong et al., 1996; Fernández et al., 2011). This mechanism has been seen mediating the resistance of *P. aeruginosa* isolates to β-lactams, aminoglycosides, polymyxins and fluoroquinolones (Zhang et al., 2001; Poole, 2005; Fernández et al., 2010; Khaledi et al., 2016).

It has been seen that once strains encounters certain concentrations of antibiotics, they can tolerate higher concentrations in subsequent exposures, while cross-resistance to other antibiotics may occur as well (Mouneimné et al., 1999; Fujimura et al., 2009; Fernández et al., 2011; Pagedar et al., 2011). Furthermore, these alterations may link to other physiological events triggered by other stimuli and stresses as well as mutations in some specific genes (Xiong et al., 1996; Karlowsky et al., 1997; Fernández et al., 2011).

Using isolates from CF patients, it was shown that adaptive resistance of *P. aeruginosa* to fluoroquinolones such as ciprofloxacin is due to multiple mutations in the known-resistance genes including the *gyrA, gyrB, nfxB*, and *orfN* which were concomitant with mutations in the genes involved in cyclic di-GMP signaling (Figure 3). Mutations of *nfxB* were prevalent leading to loss of function of NfxB transcriptional repressor and consequently leading to the overproduction of MexCD-OprJ efflux pump (Wong et al., 2012; Figure 3). This efflux pump is an important determinant of resistance to fluoroquinolone antibiotics (Hirai et al., 1987). On the other hand, another study showed that expression of the *mexCD-oprJ* genes depends on the sigma factor AlgU and leads to resistance to the biocide chlorhexidine (Fraud et al., 2008). AlgU is well-known stress response sigma factor which positively regulates overproduction of alginate in mucoid isolates (Hershberger et al., 1995).

Another group showed that *P. aeruginosa* can acquire and lose resistance in the presence and absence of colistin, respectively. This occurred via adaptive multiple mutational mechanisms and genetic reversion (Lee et al., 2016). It was also demonstrated that resistance to certain polycationic antimicrobials such as aminoglycosides, polymyxins and cationic antimicrobial peptides can be mediated by altering the lipid A structure in LPS. This was caused by multiple mutations in cognate regulatory proteins such as the two-component systems PhoP-PhoQ, PmrA-PmrB, CprR-CprS, and ParR-ParS (Barrow and Kwon, 2009; Fernández et al., 2012; Figure 3). Other studies showed that further and complex genetic alterations affecting regulatory pathways including those causing amino acid substitutions in these cognate regulatory proteins such as PhoQ and PmrB are involved in polymyxin resistance. This is why the mechanism of resistance of *P. aeruginosa* to colistin was found to vary among isolates (Lee et al., 2016). Interestingly, this study showed that the acquisition of colistin resistance via many amino acid substitutions is reversible in colistin-susceptible revertants. However, even in the absence of colistin, resistance was preserved for some time and emergence of revertants may not occur so fast (Lee et al., 2016).

### QS-dependent antibiotic resistance

Some direct and indirect evidences have been found linking the QS systems with antibiotic resistance mechanisms in *P. aeruginosa* (Rasamiravaka and El Jaziri, 2016), but further exploration is needed for better understanding. Using clinical strains of *P. aeruginosa*, it was shown that the *las* system positively links to the expression of *mexY* gene encoding the inner-membrane drug/H^+^ antiporter protein MexY (Pourmand et al., 2015) which is a key subunit of the MexXY-oprM complex known as a major determinant of aminoglycoside resistance (Lau et al., 2014). On the other hand, some studies showed that CF-infecting strains with the common *lasR* loss-of-function mutations were more resistant to therapeutic antibiotics such as tobramycin, ciprofloxacin and ceftazidime. The reported antibiotic resistances in the *lasR* mutants were attributed to increased β-lactamase activity, bacterial metabolic adaptation or metabolic shifts (D'Argenio et al., 2007; Hoffman et al., 2010). However, the relationship of antibiotics susceptibility with the *rhl* encoded QS system and production of C4-HSL signals remains unclear (Bjarnsholt et al., 2010). Some supporting evidence was obtained by, treating *P. aeruginosa* biofilms with ciprofloxacin which upregulated the production and secretion of the virulent factor LasB, which is under the control of Rhl QS system (Oldak and Trafny, 2005; Figure 1).

Furthermore, two independent studies reported that the clinical strains of *P. aeruginosa* with QS-deficient phenotypes and negative for the production of QS-dependent virulence factors could cause infections and tend to be less susceptible to antimicrobial agents (Karatuna and Yagci, 2010; Wang H. et al., 2013). However, it was not shown how these mechanisms might link to each other while many of these clinical strains could also form biofilms with antibiotic resistance traits and many regulatory pathways for biofilm development are under the control of QS systems. Zhao et al. reported some supporting information showing the importance of QS systems in both biofilm formation and antimicrobials induced expression of *ampC* (Zhao et al., 2015). Earlier studies showed that by overexpressing the chromosomal type 1 β-lactamase, QS-dependent virulence factors were reduced and strains were less virulent (Ramisse et al., 2000). Also, Kong et al. analyzed the dual role of the AmpR transcriptional regulator where it positively regulated β-lactamases and negatively regulated the virulence factors through QS systems (Kong et al., 2005).

Balasubramanian et al. analyzed co-regulatory and transcriptional networks of three co-existing mechanisms involved in β-lactam resistance, alginate production and modulation of virulence factor expression. They showed that while AmpR positively and negatively regulates β-lactamases and QS-dependent proteases, respectively, there is an intimate crosstalk between the AmpR regulon and the master regulator AlgU which positively regulates alginate production (Balasubramanian et al., 2011). This gave more insight into the complexity of such co-existing networks. Recent findings also showed that high levels of cyclic di-GMP mediated by the SagS regulator contributes to elevated antibiotic resistance via BrlR regulon-dependent upregulation of cognate genes encoding MexAB-OprM and MexEF-OprN multidrug efflux pumps (Gupta et al., 2014).

The periplasmic TpbA tyrosine phosphatase was also reported as a regulatory candidate for linking QS signaling and biofilm formation. This protein was shown to be positively regulated by the *las* QS system at transcriptional level. Upon production of TpbA and its phosphatase activity in the periplasm, the cyclic di-GMP synthesizing protein TpbB is dephosphorylated at a tyrosine residue in periplasmic domain leading to inactivation of TpbB and a reduction in cyclic di-GMP levels and in turn Pel production, hence inhibiting biofilm development (Ueda and Wood, 2009). TpbA-dependent cyclic di-GMP reduction was also linked to increasing eDNA release by cell lysis (Ueda and Wood, 2010).

Overall, the reason for inconclusive information about the relation of the QS system and antibiotic resistance mechanisms is based on the fact that there are various layers of regulatory pathways associated with both QS systems and antibiotic resistance mechanisms in *P. aeruginosa*. Therefore, understanding the interplay between hierarchical QS systems and various antibiotic resistance mechanisms needs further exploration.

## Adaptive radiation for persistence

Adaptation to the surrounding environments is an extraordinary capability of *P. aeruginosa*. It enables *P. aeruginosa* to inhabit diverse ecological niches such as colonization of various hosts as well as long term persisting infections. The adaptation process is designated as adaptive radiation by which initial clones would diversify into a variety of genotypes and phenotypes over time until the most favorable and adapted descendants are selected for long term persistence (Hogardt and Heesemann, 2010). A typical example is adaptation of *P. aeruginosa* isolates to the CF airways. Various studies have shown that initial colonization of CF lungs is caused by wild-type strains existing in the environment. For bacteria, the CF lungs encompass various stresses such as oxidative stresses and immune responses and inter-species competition followed by antibiotic treatment. Therefore, initial clones undergo substantial adaptation processes to survive such hostile environments.

**Figure 4:**
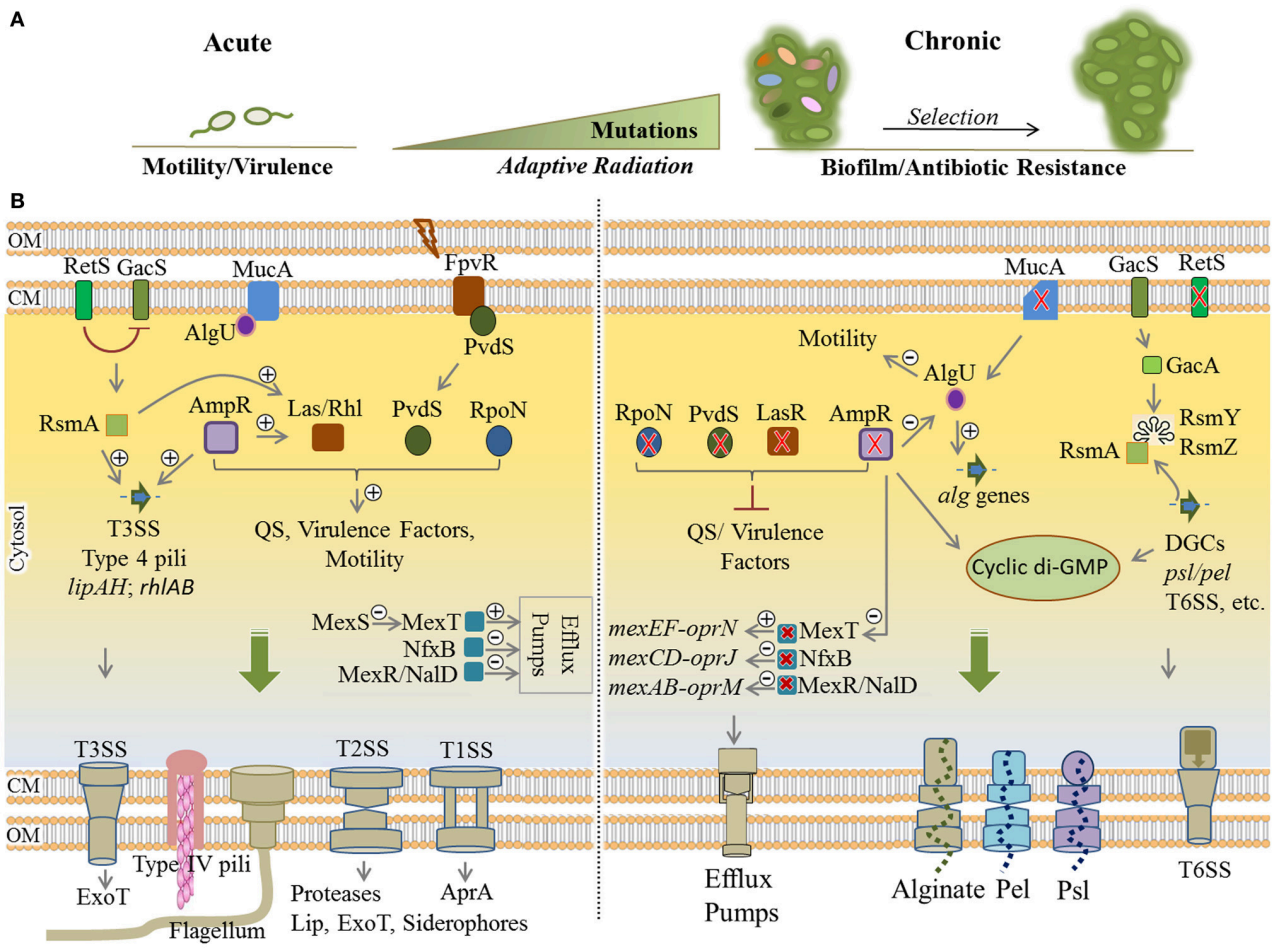
**Remodeling of regulatory networks in *P. aeruginosa* during adaptive radiation and transition from acute to chronic infections. (A)** During pathogenesis, adaptation to the CF lung environment occurs through “adaptive radiation” where intense genetic mutations lead to diverse genotypes and phenotypes (colorful ellipsoids) within bacterial populations followed by the selection of colonizers. Mutational adaptation and selection of generations drive bacterial transition from acute to chronic traits. **(B)** Remodeling of key regulatory networks between acute and chronic infections occurs mainly via mutational adaptation in cognate genes. Mutated *lasR, ampR*, and *retS* genes are key determinants in this process by which QS, virulence factor production and motility are downregulated, while synthesis of cyclic di-GMP, exopolysaccharides and various multidrug efflux pumps are upregulated. Mutation of *mucA* results in a defect in MucA (anti-sigma factor) releasing AlgU (positive regulator of alginate operon) that induces overproduction of alginate and the mucoid phenotype. Of important acute traits are flagellum, type 4 pili, T1SS, T2SS, T3SS (types 1 to 3 secretion systems), ExoT (exotoxins), Lip (lipases), AprA (alkaline proteases). The type 6 secretion system (biofilm-associated and toxin-delivering device to other bacteria) and efflux pumps and the production of EPS are part of chronic traits which confer antibiotic resistance and/or mediate biofilm formation. Plus and minus signs represent positive and negative effect of transcriptional regulators, respectively. Red cross indicates mutagenesis. CM, cytoplasmic membrane; OM, outer membrane.

Here, adaptive radiation is mainly due to intense genetic adaptations leading to the thousands of generations displaying diverse genotypes and phenotypes that emerge *in vivo*, while subjected to selection pressure imposed by the CF lung milieu (Figure 4). Therefore, selected variants display different genotypes when compared with initial wild-type colonizers and persist in the CF lungs leading to clonal expansion within patients and establishment of chronic infections (Mathee et al., 1999; Kong et al., 2005; Boles and Singh, 2008; Driffield et al., 2008; Workentine et al., 2013). By assessing a wide selection of phenotypes, Workentine et al. showed that the overall population structure in one chronically infected patient can be much more heterogeneous in phenotypes than what has been previously documented (Workentine et al., 2013). Furthermore, it has been reported that transmission of strains from patient to patient can result in the coexistence of highly divergent bacterial lineages (Winstanley et al., 2016).

Generally, phenotypic adaptation of these strains include slow growth, auxotrophy, virulence deficiency via downregulation of QS systems, loss of motility, biofilm formation, alginate overproduction and mucoid phenotype, antibiotic resistance, hypermutability, and lipopolysaccharide modifications. Downregulation of virulence factors such as flagella motility, T2SS/T3SS apparatus, and toxic components results in less inflammatory and phagocytic responses since the pathogen is less detectable for the immune system (Mahenthiralingam et al., 1994; Hogardt and Heesemann, 2010). Analysis of many clinical isolates showed these alterations represent convergent molecular evolution among many clinical isolates and mutation of 52 genes are mainly responsible for substantial phenotypic alterations associated with virulence traits and resistance (Diaz Caballero et al., 2015; Marvig et al., 2015). Of these genes, common adaptive mutations occur in regulatory genes including *lasR, pvdS, rpoN, mucA, mexT, nfxB, mexR, nalD, retS, and ampR* (Figure 4). Collectively, this leads to remodeling regulatory networks and developing a general adaptation pattern as explained below (Higgins et al., 2003; Hogardt and Heesemann, 2010; Rau et al., 2010; Winstanley et al., 2016).

Mutation of *lasR, pvdS*, and *rpoN* impairs central QS system signal processing leading to the deficiency in virulence traits. In wild-type strains, the LasR and PvdS regulators control the expression of a large number of genes including key virulence factors (Table 1, Figure 4) and pyoverdine for iron acquisition, respectively (Table 1; Hoffman et al., 2009; Imperi et al., 2010; Jiricny et al., 2014; LaFayette et al., 2015). The alternative sigma factor RpoN has been also found to regulate many cell functions such as motility and virulence factors production via QS system (Cai et al., 2015; Figure 4). Additionally, knocking out mutation of *mucA* locus encoding anti-sigma factor MucA results in releasing the RNA polymerase sigma factor σ^22^ (AlgU) which itself positively regulates expression of the alginate biosynthesis operon and stress response mechanisms while it negatively regulates several virulence factors such as flagella, pili, T3SS, and Rhl quorum sensing signals (Folkesson et al., 2012). Development of the mucoid phenotype mediated by alginate overproduction as well as the formation of highly structured biofilms is the hallmark of chronic infections (Schurr et al., 1996; Hay I. D. et al., 2013; Figure 4).

Another common mutation has been reported in the *mexT* locus resulting in activation of MexT, the positive regulator of MexEF-OprN efflux pump, which in turn led to antibiotic resistance. In addition, the induction of MexEF-oprN production is linked to extruding QS signaling molecules and reduction of virulence factor production (Tian et al., 2009; Figure 4). The MexEF-OprN production is undetectable in wild-type strains due to the non-functionality of the *mexT* gene (Maseda et al., 2000, 2010). Furthermore, mutations of repressor genes *nfxB* and *mexR/nalD* in clinical strains upregulated the production of the MexCD-OprJ and MexAB-OprM efflux pumps, respectively, conferring resistance to a wider range of antibiotics (Higgins et al., 2003; Sobel et al., 2005; Jeannot et al., 2008; Rau et al., 2010; Figure 4). Also, mutation of other genes such as *gyrA*/*gyrB* (DNA gyrase), *mexZ* (transcriptional regulator of the *mexXY*) and *mexS* (transcriptional regulator of the *mexEF*) are commonly attributed to antibiotic resistance during mutational adaptations (Marvig et al., 2015; Figure 4).

As part of RetS/GacS/GacA/RsmA regulatory pathway, the *retS* gene is important for phenotypic shifting from acute to chronic infections (Lapouge et al., 2008; Moscoso et al., 2011). The *retS* mutation repressed the production of virulence factors such as T3SS and swarming motility while it upregulated production of the T6SS (type 6 secretion system) and exopolysaccharides Pel/Psl required for biofilm formation (Moscoso et al., 2011; Figure 4). The T6SS is a puncturing device for delivery of proteins and toxins into the competing bacteria and the host cells and an important survival advantage for *P. aeruginosa*. It is also required for biofilm formation while being considered as a virulence factor (Chen et al., 2015). This transitional impact was shown to be mediated by high levels of cyclic di-GMP (Boehm et al., 2010; Paul et al., 2010; Moscoso et al., 2011).

Mutation of the *ampR* gene is another common mutational adaptation with a large impact on remodeling of physiological traits (Figure 4). The AmpR global regulator in *P. aeruginosa* regulates not only resistance to different classes of clinically relevant antibiotics, but also expression of hundreds of genes involved in diverse physiological processes such as virulence, QS systems and stress responses (Balasubramanian et al., 2015). It is understood that the *ampR* mutation induces adaptations leading to chronic infection including the downregulation of stress responses and virulence factors via downregulating QS systems, and boosting biofilm formation and alginate overproduction by causing elevation of cyclic di-GMP levels. Additionally, it induced AlgU activity, and resistance to fluoroquinolone through activation of MexT upregulating the MexEF-OprN efflux pump as well as increasing twitching motility and T6SS production (Balasubramanian et al., 2011, 2012, 2014, 2015; Figure 4).

Other adaptive mutations of CF isolates have been commonly reported in anti-mutator genes including *mutS, mutT, mutL, mutY, mutM*, and *uvrD* conferring a “hypermutability phenotype” with elevated mutation rates due to the lack of DNA repair mechanisms. This phenotype has been described as being caused by later mutational events as they are understood to occur after mutation of the *lasR* and *mucA* genes known as earlier mutations. However, other reports postulated that mutation of anti-mutator loci may increase the rate of other adaptive mutations (Oliver and Mena, 2010).

Hypermutators are very prevalent in CF isolates and they are shown to have correlation with higher antibiotic resistance particularly in the late stage of chronic infections. However, hypermutators also display other phenotypes such as mucoidity, lack of motility and LPS production (Oliver et al., 2000; Ciofu et al., 2010; Varga et al., 2015).

Other distinct phenotypes correlated with adaptation to CF airways are the small-colony variants (SCVs). They are associated with prolonged persistence and chronic infections in CF lungs and obstructive pulmonary diseases (Malone, 2015). They have been characterized as variants forming rugose small colonies on solid media (1–3 mm in diameter) with slow growing, autoaggregative and enhanced biofilm formation characteristics combined with enhanced surface attachment and hyperpiliation for twitching (Häussler et al., 1999, 2003; Kirisits et al., 2005). *In vitro* analyses showed that the SCVs display increased resistance to a wide range of antibiotics (Wei et al., 2011). Different studies have demonstrated that the presence of SCVs in the CF lung is associated with poorer lung function and clinical outcomes (Häussler et al., 1999, 2003; Schneider et al., 2008).

It has been understood that SCVs show high levels of cyclic di-GMP production aligned with increased production of Pel and Psl exopolysaccharides (Starkey et al., 2009). So far investigations regarding the molecular mechanisms underlying SCVs formation confirmed loss-of-function mutations in regulatory genes such as *wspF, yfiR* and *rsmA* and some other genes which alter regulatory networks in favor of enhanced cyclic di-GMP production (Irie et al., 2010; Malone et al., 2012; Blanka et al., 2015; Malone, 2015). On the other hand, an upregulating cyclic di-GMP synthesis pathway is a key determinant of exopolysaccharide production leading to highly developed biofilms. However, it still remains unclear how two distinct phenotypes i.e., cell within mucoid biofilm and SCV differ in regard to the cyclic di-GMP mediated signaling pathways.

## Survival by stringent response and persister formation

Stringent response to environmental stresses such as nutritional starvation and response to antibiotics and oxidative stresses share a similar outcome of adaptation i.e., all are leading to dormancy and persister formation. In both responses, bacteria slow down their metabolism through downregulating the expression of genes participating in the biosynthesis of proteins, ribosomes, cell wall, nucleic acid metabolism, and virulence factors. These dramatic metabolic alterations result in arresting cell growth and cell division in favor of bacterial survival (Eymann et al., 2002; Hesketh et al., 2007; Durfee et al., 2008).

Persisters are defined as subpopulations of cells, occurring at very low frequency, which stochastically emerge in the presence of stress. They show very slow growth enhancing survival under stress while viability of the majority of the population is severely impaired. Upon stress removal, persisters turn back to normal growth to propagate, which coincides with regained sensitivity to stress. Such persistence was suggested to be based on the heterogeneity of population by means of epigenetic mechanisms, not genetic mutations (Fasani and Savageau, 2015).

Various studies have provided evidences showing the link between stringent response and persistence, but mostly using *E. coli* as a model which can be informative for *P. aeruginosa* as it possesses homologous signaling pathways (Fung et al., 2010; Maisonneuve et al., 2013; Amato et al., 2014; Ramisetty et al., 2016). There are only a few studies aiming to explain such responses, but they provided inconclusive explanations. Therefore, we summarized the general findings in order to propose the underlying molecular mechanisms in *P. aeruginosa*.

### Molecular mechanisms underlying stringent responses and persisters

Notably, increased levels of (p)ppGpp (collectively designated for guanosine pentaphosphate and guanosine tetraphosphate) molecules in the cells is a central triggering alarmone for both persistence and stringent response (Potrykus and Cashel, 2008; Wu et al., 2010; Amato et al., 2014). The cellular levels of (p)ppGpp are mediated by the activity of the (p)ppGpp-synthesizing and degrading enzymes such as RelA and SpoT in response to external stimuli (Bremer and Dennis, 2008).

In stringent response when *E. coli* encounters amino acid deprivation, the ribosome-associated RelA synthesizes ppGpp molecules to an upper level. In association with the transcriptional regulator DksA (global regulator of metabolism), ppGpp interacts with RNA polymerase and inhibits the transcription of ribosomal RNA promoters. This inhibitory impact is concomitant with activation and upregulation of pathways for amino acid biosynthesis and the transcription of stress response genes (Potrykus and Cashel, 2008; Dalebroux and Swanson, 2012; Amato et al., 2014). Amato et al. (2013) found that stringent responses are linked to the emergence of persisters by involving ppGpp based regulatory events (Amato et al., 2013).

The persister state is typically based on the activity of genetically encoded toxin-antitoxin (TA) modules particularly in response to antibiotics, but proposed as being activated by the same acting elements i.e., RelA or SpoT and (p)ppGpp in *E. coli* (Maisonneuve et al., 2013). These TA system are widely distributed in genomes or plasmids of bacteria and archaea (Van Melderen, 2010). Basically, the toxin element is a stable protein while the cognate antitoxin element either is a protein or a small RNA molecule which are metabolically unstable under unfavorable conditions. A small RNA antitoxin directly inhibits the toxin translation by pairing with toxin mRNA or inactivate toxin by direct binding. A protein antitoxin on the other hand either degrades toxin mRNA or blocks the activity of cognate protein toxin via direct protein-protein interaction or protective interaction with toxin substrates. Impairment of antitoxin function under certain stresses such as antibiotics as well as nutritional stresses leads to the accumulation and activation of toxin proteins. Consequently, the toxin targets and interferes with key cellular processes such as DNA replication, and the synthesis of tRNA, membrane components, and ATP leading to the inhibition of cell growth and cell division to form a dormant or persister cell (Christensen-Dalsgaard et al., 2010; Wen et al., 2014). Presumably, bacteria switch off their active metabolism upon exposure to stress to evade starvation or antibiotic impact on their cellular targets. Christensen et al. demonstrated that *E. coli* encodes at least 10 TA loci and all of which could be induced by nutritional starvation and antibiotics (Christensen et al., 2001; Christensen-Dalsgaard et al., 2010).

**Figure 5:**
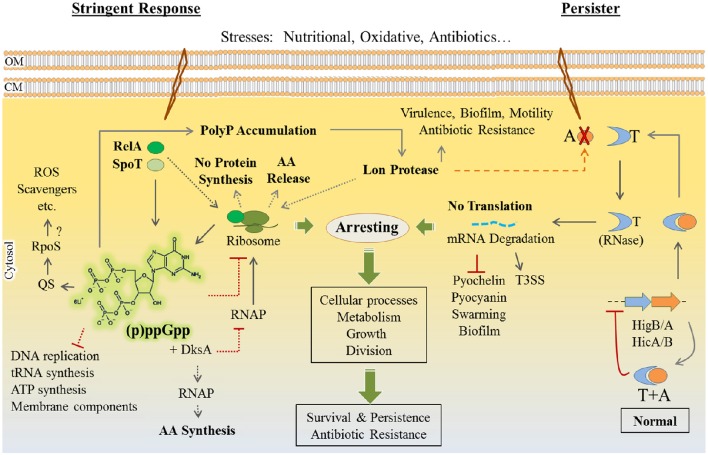
***P. aeruginosa* stringent response and persister formation**. Stringent response is triggered by particular stresses such as amino acid and fatty acid starvation, iron/phosphor depletion and oxidative stress [e.g., reactive oxygen species (ROS)]. The (p)ppGpp alarmone is a key determinant for stringent response and it is elevated by RelA/SpoT enzymes. Generally, (p)ppGpp elevation and the PolyP (inorganic polyphosphate) and Lon protease complex interfere with normal biological processes in favor of bacterial survival via arresting metabolism, cell growth and cell division (dashed gray pathways are best understood for the *E. coli* model, but not or partially characterized in *P. aeruginosa*). In *E.coli*, (p)ppGpp signaling is linked to toxin (T)-antitoxin (A) system via activation of the Lon protease leading to the formation of persisters displaying dormant and antibiotic resistance phenotypes (dashed orange line). Generally, the TA complex is stable under normal conditions suppressing toxin activity and further expression of cognate genes. Upon antitoxin degradation, toxin becomes active to hinder biological processes. In the case of *P. aeruginosa* HigB/A, HicA/B, the toxin components perform endoribonuclease (RNase) activity on mRNA molecules. In *P. aeruginosa*, the (p)ppGpp alarmone is linked to the production of ROS scavengers probably via QS or RpoS regulators and Lon activity is required for biofilm formation, motility, virulence and antibiotic resistance. Furthermore, the TA system downregulates biofilm formation and virulence factor production while T3SS (type 3 secretion system) can be found upregulated. Although, the (p)ppGpp signaling, Lon protease activity and TA modules (i.e., HigB/A, HicA/B, and likely more complexes) are present in *P. aeruginosa*, their link to resistance to antibiotics and other stresses is poorly understood. AA, amino acids; QS, quorum sensing; RNAP, RNA polymerase. CM, cytoplasmic membrane; OM, outer membrane.

So far, Maisonneuve et al. (2013) have proposed the best model presenting the hierarchical molecular mechanisms for *E. coli* persistence with the involvement of the key players including RelA/SpoT enzymes, (p)ppGpp signaling, Lon protease (ATP-dependent protease), inorganic polyphosphate (PolyP), and toxin-antitoxins systems (Maisonneuve et al., 2013; Figure 5). This model explains that (p)ppGpp synthesized by RelA/SpoT inhibits PolyP degradation, while PolyP accumulation stimulates Lon protease to degrade the antitoxin leading to the activation of toxin for arresting cellular processes and growth. Previously, it was shown in *E. coli* that in response to nutritional stress and antibiotics, transcriptional activation of TA loci depended on protease activity of Lon protease on antitoxin which itself repressed the TA promoter (Christensen-Dalsgaard et al., 2010). Furthermore, it was suggested that the complex of Lon protease with PolyP could promote ribosomal protein degradation for supplying required amino acids during starvation (Kuroda et al., 2001; Figure 5).

### *P. aeruginosa* stringent response and persisters

Required elements of stringent response and persistence including the ppGpp alarmone, SpoT, RelA, DksA, and the TA modules have been characterized in *P. aeruginosa* (Figure 5). Mutants deficient in *relA* and/or *spoT* genes showed increased sensitivity to heat shock, oxidative and osmotic stresses as well as antibiotics while becoming less virulent. Stringent response and RelA/SpoT activity for production of ppGpp were found to be crucial for regulation of virulence factor production (Erickson et al., 2004; Viducic et al., 2006; Boes et al., 2008; Nguyen et al., 2011; Vogt et al., 2011). Also, there is some experimental support that *P. aeruginosa* utilizes stringent response to protect cells from oxidative stresses generated by toxic reactive oxygen species (ROS) under aerobic conditions. The mutants deficient in *relA* and *spoT* genes were highly susceptible to multiple oxidants. This study showed that (p)ppGpp signaling is necessary for optimal expression of catalase and superoxide dismutase enzymes as major ROS scavengers, but it was assumed to be indirectly regulated through a complex regulatory network (Sampathkumar et al., 2016; Figure 5). Because, (p)ppGpp signaling is also required for full expression of other regulatory pathways controlling antioxidant response such as the stress response regulator RpoS as well as both Las and Rhl QS systems (van Delden et al., 2001; Kohanski et al., 2008; Schafhauser et al., 2014; Sampathkumar et al., 2016; Figure 5). The proposed *E. coli* model for the activation of the TA system via Lon protease has not been shown for *P. aeruginosa*, yet. However, different studies indicated that Lon protease activity was induced by aminoglycosides and *lon* mutants were highly susceptible to ciprofloxacin while *lon* was also required for biofilm formation, motility and virulence (Marr et al., 2007; Breidenstein et al., 2012). Molecular mechanism of these pathways have not been well characterized, but as proposed previously, interconnection of various regulatory and signaling pathways for appropriate responses leading to either as stringent/persistence, biofilm formation or virulence is anticipated (Kim et al., 1998).

To date five types (I-V) of TA systems have been described in bacteria based on the nature and mode of action of antitoxin (Wang et al., 2012). So far, HigB/HigA and HicA/HicB TA modules encoded by genomic loci have been experimentally demonstrated in *P. aeruginosa*, while other TA systems such as the *relBE* and *parDE* loci were predicted but they have not been characterized, yet (Pandey and Gerdes, 2005; Fernández-García et al., 2016; Figure 5). The HigB/HigA and HicA/HicB TA modules have been also widely reported for other bacteria (Pandey and Gerdes, 2005; Li G. et al., 2016; Wood and Wood, 2016).

These TA modules belong to the type II TA system where both toxin (i.e., HigB/HicA) and antitoxin (i.e., HigA/HicB) are proteins directly interacting with each other retaining the toxin inactivated, such as inhibiting the RNase activity of HigB or HicA (Christensen et al., 2001; Rocker and Meinhart, 2016; Figure 5). The HigB/HigA TA module was found to influence *P. aeruginosa* pathogenicity where toxin HigB was shown to reduce the production of the virulence factors pyochelin, pyocyanin, swarming, and impaired biofilm formation representing a novel function for a TA systems (Wood and Wood, 2016; Figure 5). Another study showed that the HigB/HigA TA module was necessary for the ciprofloxacin induced persister formation by *P. aeruginosa*. Concurrently, HigB overproduction upregulated the expression of T3SS genes leading to the accumulation of T3SS proteins in persisters as well as increasing bacterial cytotoxicity against host immune cells (Li M.et al., 2016; Figure 5).

Furthermore, these TA systems have been shown to be highly prevalent in the clinical strains (Pandey and Gerdes, 2005; Williams et al., 2011; Li G. et al., 2016). It is believed that persisters are one of the main reasons for recurring and chronic infections where persisters withstand antibiotic treatments and spawn new infecting population upon removal of antibiotic treatment (Lewis, 2007; Wang and Wood, 2011). They are abundant in *P. aeruginosa* biofilms which is the hallmark of long-term infections particularly in CF patients (Lewis, 2008; Mulcahy et al., 2010).

## *P. aeruginosa* resistance to foreign DNA

Infection of bacteria with viruses or bacteriophages is a natural phenomenon which can lead to bacterial lysis. Bacteria harness various mechanisms to destroy such foreign DNAs leading to resistance. The CRISPR (clustered regularly interspaced short palindromic repeats)-Cas (CRISPR associated proteins) systems form the only adaptive immune system in prokaryotic cells which also mediates *P. aeruginosa* survival during viral invasions (Cady et al., 2012; Bondy-Denomy and Davidson, 2014). A CRISPR region is an array of multiple repeated sequences on the bacterial genome or a plasmid ranging from 21 to 48 bp in length and separated by 26 to 72 bp hypervariable spacers (Bhaya et al., 2011; Cady et al., 2012). The *cas* locus encoding Cas proteins is located in the vicinity of the CRISPR region (Bhaya et al., 2011). In principle, the molecular mechanism is based on acquisition and integration of small fragments of foreign DNAs such as derived from viruses into the spacer regions between two adjacent repeats within the CRISPR locus mediated by Cas proteins with nuclease activity. Subsequently, the CRISPR region is transcribed resulting in pre-CRISPR RNA (pre-crRNA) which undergoes hydrolysis by endoribonucleases forming small CRISPR RNAs (crRNAs). The mature crRNAs in association with a multiprotein complex known as CASCADE (CRISPR-associated complex for antiviral defense) recognizes invasive DNAs upon complementarity which results in the initiation of the cleavage of the crRNA–foreign DNA hybridization complex, mediating survival of bacteria after viral infections while protecting themselves from lysis (Brouns et al., 2008; Mojica et al., 2009; Deveau et al., 2010; Garneau et al., 2010; Bhaya et al., 2011). The CRISPR-Cas systems have been classified into three major types (I, II and III) and at least 11 subtypes (IA-F, IIA-C and IIIA-B) encoding distinct crRNA-guided surveillance complexes (Makarova et al., 2011).

A study showed that 36% of tested *P. aeruginosa* clinical isolates harbored CRISPR-Cas systems developing adaptive immunity against various mobile genetic elements such as temperate phages, prophages, pathogenicity island transposons which were integrated into the genome (Cady et al., 2011). Different studies demonstrated that the types I-F and I-E CRISPR-Cas systems are naturally active in *P. aeruginosa* isolates (Cady et al., 2012; Pawluk et al., 2014). A recent phylogenetic study revealed the existence of the type I-C CRISPR-Cas system in some isolates of *P. aeruginosa* (van Belkum et al., 2015).

On the other hand, the activity of CRISPR/Cas system can be inhibited by anti-CRISPR/Cas genes harbored by phages infecting *P. aeruginosa* which counteract the type I-F and I-E systems (Bondy-Denomy et al., 2015; Maxwell, 2016). Also it has been shown that phages producing anti-CRISPR activity are closely related to each other and with high sequence similarity to bacteriophage DMS3 (Bondy-Denomy et al., 2013). Bacteriophage DMS3 was isolated from clinical isolates of *P. aeruginosa* and it was shown to inhibit biofilm formation and swarming motility, and *P. aeruginosa* cannot develop immunity against it due to the lack of complementarity between crRNA and protospacers of DMS3 genome (Budzik et al., 2004; Zegans et al., 2009). Furthermore, the CRISPR-Cas systems show a strong correlation with antibiotic resistance/susceptibility (van Belkum et al., 2015). Additionally, the same study showed that the CRISPR-Cas systems play an important role in shaping the accessory genomes of globally distributed *P. aeruginosa* strains. Accessory genome is referred to highly variable regions of the genome versus a relatively invariable core genome. *P. aeruginosa* accessory genome varies from strain to strain, ranging from 6.9 to 18.0% of the total genome, and is mainly comprised of integrative and conjugative elements, replacement islands, prophages and phage-like elements, transposons, insertion sequences and integrons (Kung et al., 2010; Ozer et al., 2014). According to this finding the CRISPR typing with regard to the frequency of spacer integration and deletion between related strains can potentially be used for identifying the lineage of strains especially within outbreaks (van Belkum et al., 2015).

Overall, understanding of the CRISPR-Cas and anti-CRISPR-Cas systems is gradually becoming important in the context of pathogenesis and strain lineage identification. These links were highlighted by the discovery of the interaction of bacteriophage DMS3 and the type I-F CRISPR, and its impact on biofilms (Zegans et al., 2009; Palmer and Whiteley, 2011) as well as the role of different CRISPR/Cas systems on virulence and antibiotic resistance (Louwen et al., 2014). Overall, these findings suggest a more diverse function of CRISPR/Cas systems within the context of pathogenesis, requiring further in depth studies to elucidate the underlying molecular mechanisms.

## Conclusions and perspectives

For many years, *P. aeruginosa* has been a model organism and received much attention from scientific community to study the bacterial lifestyle and pathogenesis. It always has been of particular importance due to causing persistent infections in CF and immunocompromised patients. Nowadays, this ubiquitous bacterial pathogen is accepted worldwide as a public health risk due to its increasing prevalence in healthcare acquired infections combined with its ability to develop resistances to multiple classes of antibiotics. Over the past decade, extensive research studies have focused on these growing concerns aiming at deciphering the nature of *P. aeruginosa* capability and underlying molecular mechanisms applying different modes of persistence and antibiotic resistance.

In this review, we summarized several of the well characterized molecular mechanisms which enable *P. aeruginosa* to survive various hostile conditions such as during pathogenesis and antibiotic treatment. These mechanisms form multiple layers of physiological adaptations correlating with social behavior and lifestyle of bacteria while responding environmental stimuli. Such extraordinary adaptive capability relies on extensive numbers of regulatory or controlling factors within integrated and complex signal processing pathways. These enable bacteria perceive and process environmental cues in order orchestrate physiological changes to promote adaptation to unfavorable conditions. Many of these regulatory pathways, their cognate player, their signals and how they are integrated with global regulatory networks still remain poorly understood.

Furthermore, we highlighted key molecular pathways driving *P. aeruginosa* survival and persistence at different stages of pathogenesis such as QS elements for virulence traits, cyclic di-GMP signaling in biofilm formation and development of chronic traits, (p)ppGpp signaling/TA system in persister formation and various strategic adaptations for developing resistance to divers classes of antibiotics.

Recent technological advances in genomic characterization of pathogens have provided invaluable information about the dynamics of *P. aeruginosa* populations and their heterogeneity at different stages of pathogenesis. These results which were explained under “adaptive radiation” term emphasized that this species shows a stunning capability to become resilient during pathogenesis to withstand antibacterial treatment.

Available information indicated that sole therapy which only relies on bacteriostatic/bactericidal compounds can readily be defeated by bacterial resiliency and a management program is still required to combat infections. This program should be able to predict and evaluate physiological adaptations at each stage of infection for exerting appropriate treatments which could interfere with adaptation rather than increasing the chance of bacterial survival. A good example is improper and frequent application of antibiotics which must be avoided. Instead a comprehensive hygiene program must be applied in healthcare settings and among personnel to stop the spread of nosocomial infections specifically caused by multidrug resistance strains. Also, further research on identification of new drugs and developing new alternative prevention and treatment strategies for interfering with key regulatory pathways is needed.

At the end we suggest all efforts should consider international coordinated multidisciplinary programs with results of laboratory outputs being deposited in centralized accessible databases to expedite advances in control of infections and its implementation into clinical settings. The steadily growing concern of emerging antibiotic resistance strains in the world, would justify the set-up of such databases which then allow developing world-wide guidelines for monitoring and recording antibiotic resistance cases around the world. This should provide healthcare experts with appropriate guidelines for well managing bacterial infections and preventing the rate and spread of resistance strains.

## Author contributions

MM and BR conceived and wrote the majority of the manuscript. SG contributed biofilm and alginate related aspects to the manuscript.

## Funding

The work was funded via the Massey University Research fund.

### Conflict of interest statement

The authors declare that the research was conducted in the absence of any commercial or financial relationships that could be construed as a potential conflict of interest.
